# Characterization and mapping of *Dt1* locus which co-segregates with *CcTFL1* for growth habit in pigeonpea

**DOI:** 10.1007/s00122-017-2924-2

**Published:** 2017-05-24

**Authors:** Rachit K. Saxena, Jimmy Obala, Andrey Sinjushin, C.V. Sameer Kumar, K.B. Saxena, Rajeev K. Varshney

**Affiliations:** 1 International Crops Research Institute for the Semi-Arid Tropics (ICRISAT), Patancheru, 502324 India; 2 University of KwaZulu-Natal, African Center for Crop Improvement, Private Bag X01, Scottsville, 3209 Pietermaritzburg, South Africa; 3 M. V. Lomonosov Moscow State University, Leninskie Gory, Moscow, 119991 Russia

## Abstract

*****Key message***:**

**We report growth habit profiling following SEM, genetic mapping and QTL analysis. Highlighted** ***CcTFL1*** **, a candidate for determinacy in pigeonpea, since an Indel marker derived from this gene co-segregated with** ***Dt1*** **locus.**

**Abstract:**

Pigeonpea (*Cajanus cajan*) is one of the most important legume crops grown in arid and semi-arid regions of the world. It is characterized with few unique features compared with other legume species, such as *Lotus*, *Medicago*, and *Glycine*. One of them is growth habit, an important agronomic trait. In the present study, identification of mutations affecting growth habit accompanied by a precise analysis of phenotype has been done which will shed more light upon developmental regulation in pigeonpea. A genetic study was conducted to examine the inheritance of growth habit and a genotyping by sequencing (GBS)-based genetic map constructed using F_2_ mapping population derived from crossing parents ICP 5529 and ICP 11605. Inheritance studies clearly demonstrated the dominance of indeterminate (IDT) growth habit over determinate (DT) growth habit in F_2_ and F_2:3_ progenies. A total of 787 SNP markers were mapped in the genetic map of 1454 cM map length. Growth habit locus (*Dt1*) was mapped on the CcLG03 contributing more than 61% of total phenotypic variations. Subsequently, QTL analysis highlighted one gene, *CcTFL1*, as a candidate for determinacy in pigeonpea, since an Indel marker derived from this gene co-segregated with the *Dt1* locus. Ability of this Indel-derived marker to differentiate DT/IDT lines was also validated on 262 pigeonpea lines. This study clearly demonstrated that *CcTFL1* is a candidate gene for growth habit in pigeonpea and a user-friendly marker was developed in the present study which will allow low-cost genotyping without need of automation.

**Electronic supplementary material:**

The online version of this article (doi:10.1007/s00122-017-2924-2) contains supplementary material, which is available to authorized users.

## Introduction

The growth habit can differ considerably among cultivars for a given crop. Some cultivars have a vegetative terminal bud, which allows the genotype to grow in height and spread under adequate conditions. Such habit is known as an ‘indeterminate’ (IDT) type. The other form of growth habit is designated as ‘determinate’ (DT) type. The determinate genotypes are relatively short in stature and possess reproductive terminal buds. Such genotypes when reach flowering stop growing in height. Pigeonpea is an important pulse crop which has gained economic importance in developing world, especially in Asia and Africa due to its continuous increase in demand. It is suggested that pigeonpea was domesticated from its wild species relative, *Cajanus cajanifolius* approximately >3500 years ago (Royes 1976). In wild relative species of pigeonpea, the growth habit is generally indeterminate and the genotype flowers under short-day conditions. Whereas, variability for growth habit exists in the case of cultivated pigeonpea, i.e., some genotypes are IDT type and some genotypes are DT type. On the other hand, some genotypes are also intermediate between the IDT and DT types. These genotypes are called semi-determinate (SDT) types that ultimately contribute directly to traits such as grain yield, harvesting, and seed production. Both the major types of growth habits have their own advantages and limitations in pigeonpea. For instance, DT type genotypes mature rapidly and have a shortened flowering period, allowing earlier maturity and ease of mechanical harvesting. Whereas, control of damage in the case of insect attacks, higher seed rate, etc. is among the limiting factors in popularization of DT genotypes. The DT growth habit in pigeonpea was reported controlled by a single recessive gene (Kapoor and Gupta 1991). Alternative growth habit, i.e., IDT, is a dominant trait and has many advantages which are preferred by pigeonpea growers such as multiple branching, higher yields, easy insect control, lower seed rate, low inputs, etc. However, tall habit of IDT phenotypes and continuous flowering further hampered the mechanization efforts and synchronized harvesting.

The growth habit has been studied in a number of plant species to understand the genetic and genomic controls. Identification of genetic loci underlying growth habit will allow a better understanding of the domestication process and will enable faster, manipulation of growth habit, and flowering time in future breeding efforts. Like many traits, detailed characterization for growth habit at genomic level was initiated in *Arabidopsis,* and later, orthologous genes were studied in other crops species such as soybean (Tian et al. 2010), pea (Foucher et al. 2003), common bean (Kwak et al. 2008), pigeonpea (Mir et al. 2014), etc. In *Arabidopsis*, two opposing pathways exist including genes *APETALA1* (*AP1*) which activate flowering initiation and *TERMINAL FLOWER1* (*TFL1*) as a repressor for floral initiation. Previously candidacy of homologous genes from the above-mentioned pathways was tested in other crop species following genetic linkage analysis, candidate gene association analysis, heterologous transformation, etc. (Kwak et al. 2008; Liu et al. 2010; Repinski et al. 2012; Tian et al. 2010; Foucher et al. 2003). In the case of pigeonpea, few markers associated with determinacy were identified (Mir et al. 2012), and following candidate gene analysis *CcTFL1,* a homolog of *Arabidopsis* gene *TFL1* was reported as likely candidate gene for growth habit (Mir et al. 2014).

To characterize growth habit in pigeonpea, we have followed a systematic approach which includes microscopic characterization of mutations affecting inflorescence ontogeny, understanding the inheritance of growth habit, genotyping by sequencing (GBS)-based genetic mapping, and validation of identified associated genomic segments in diverse genetic stocks. This study also presents validation to support our hypothesis that *CcTFL1* is the gene underlying growth habit in pigeonpea. In addition, a user-friendly Indel marker has been developed from *CcTFL1* for quick and accurate manipulation of growth habit in pigeonpea breeding program.

## Materials and methods

### Morphological characterization and scanning electron microscopy

The following genotypes were studied for morphological descriptions: ICPL 20338, ICPL 87091 (determinate), ICPL 20325, and ICPL 88039 (indeterminate). Plants were grown in glasshouse conditions. In addition, line ICP 7035 and some related genera were observed in the pigeonpea wild relatives’ germplasm collection. Plant material (apices and floral buds) was fixed and stored in 70% ethanol. For further scanning electronic microscope (SEM) analysis, the specimens were dissected under a stereomicroscope, critical-point dried, mounted on metal stubs and coated with Au + Pd in a sputter coater Eiko IB-3. Specimens were visualized using an SEM CamScan-S2 (Cambridge Instruments, UK; secondary electron image regime) with accelerating voltage of 20 kV.

### Field experiment and phenotyping

One F_2_ mapping population consisting of 202 individuals segregating for growth habit (DT/IDT) was developed by crossing parental lines, namely, ICP 5529 (IDT) and ICP 11605 (DT). The sowing was done in 4-m-long rows with row-to-row and plant-to-plant spacing of 75 and 30 cm, respectively. The F_3_ seeds derived from the F_2_ plants were also sown in the field in progeny test rows. F_3_ seeds from F_2_ plants were sown in a single 4-m-long row. Plants were phenotyped on single plant basis for indeterminate or determinate growth habit after the onset of flowering as described in Mir et al. (2014).

### DNA isolation and genotyping by sequencing (GBS)

Total genomic DNA was isolated from 188 F_2_s and parents’ seedlings 2 weeks after germination using NucleoSpin plant extraction kit (MACHEREY–NAGEL, USA). DNA was also isolated from a set of 262 genotypes for marker validation. The quality and quantity of DNA were checked on 0.8% agarose gel. GBS approach was used for single nucleotide polymorphism (SNP) discovery and genotyping the F_2_ population. In summary, 10 ng of total genomic DNA from each sample was digested using *Ape*KI endonuclease. The digested DNA was ligated with barcoded adaptors using T4 DNA ligase. Ligated products from each sample were mixed in equal proportion to construct the GBS libraries. Furthermore, GBS libraries were amplified, purified, and used for sequencing on HiSeq 2500 platform (Illumina Inc, San Diego, CA, USA) as mentioned in Jaganathan et al. (2015).

### SNP genotyping

The sequence reads generated on HiSeq 2500 were used for SNP identification and genotyping using GBS analysis pipeline implemented in TASSEL v4.0. The barcode containing reads were sorted, de-multiplexed, and trimmed to first 64 bases starting from enzyme cut site. Reads containing ‘N’ within first 64 bases were discarded. The good quality reads (called as tags) were aligned against the draft genome sequence of pigeonpea (Varshney et al. 2012) using Burrows–Wheeler Alignment tool (BWA) (Li and Durbin 2009). The alignment file was processed through GBS analysis pipeline for SNP calling and genotyping. SNPs with contrasting alleles in parental genotypes and having <30% missing data were retained for study. Furthermore, imputation of missing data was carried out using FSFHap algorithm implemented in TASSEL v4.0. The imputed SNPs were subjected to minor allele frequency (MAF) cutoff of 0.2 to remove missing data and such filtered SNPs were used for genetic mapping and QTL analysis.

### Primer design and polymerase chain reaction (PCR)

Information on *CcTFL1* gene was obtained from Mir et al. (2014). To find sequence variations, *CcFTL1* gene sequence was aligned onto crossing parental of mapping population, i.e., ICP 5529 (IDT) and ICP 11605 (DT) using Asha reference genome sequence. A 10 bp deletion was identified on CcLG03 at position 20698771 bp in DT genotype as compared to IDT genotype. One primer pair was designed using the online primer design software Primer3 plus. The primer pair used for amplification include: CcLG03_ 20698771_F (CAT GGC CAT TGT AGA CTT GCT (21 bp) and CcLG03_ 20698771_R (TCA CAG CAG GAT CAT CGA GT (20 bp).

PCR reaction was conducted in a total volume of 30 μL containing 21.9 μL of ddH_2_0, 2.0 μL of 10 × KAPA Taq polymerase buffer (containing 25 mM MgCl_2_), 2.0 μL of 10 mM dNTPs, 10 pmol/μL of each of the forward and reverse primers, 0.06 μL of KAPA Taq polymerase, and 2.0 μL of 20 ng/μL gDNA. A touch-down PCR was used as follows: initial denaturation at 95 °C for 5 min followed by two annealing cycles: (1) 5 cycles consisting of 94 °C for 15 s, 62 °C for 20 s, and 72 °C for 30 s and (2) 35 cycles consisting of 94 °C for 15 s, 54 °C for 30 s, and 72 °C for 30 s and a final extension at 72 °C for 20 min and hold at 4 °C for infinity. PCR products were run in 3.5% Nusieve agarose gel. Gels were stained with ethidium bromide and visualized under UV light in a transilluminator.

### Data analysis

The F_2_ and F_3_ phenotypic segregation data were subjected to Chi-square goodness-of-fit analysis to test conformation to expected Mendelian segregation ratios. Single marker regression analysis was carried out using F_2_ phenotypes as dependent variables and the F_2_ marker genotypes as independent variables in Microsoft Excel 2013. The *CcTFL1* gene marker data were incorporated into a GBS-derived SNP-based genetic map using JoinMap 4.1 with the following settings: independence LOD 2.5 to 10.0, regression mapping algorithm, recombination frequency ≥0.49 and a *χ* ^2^ jump threshold for removal of loci set 5.0, and a ‘‘Ripple’’ after adding one marker into the map. The inter-marker distances calculated from the JoinMap program were used to construct a genetic map which was displayed using MAPCHART version 2.2. Inclusive composite interval mapping (ICIM) was conducted using the version 4.0 of QTL IciMapping software.

## Results

### Scanning electron microscopy-based characterization of IDT and DT genotypes

#### Stem architecture

**Fig. 1:**
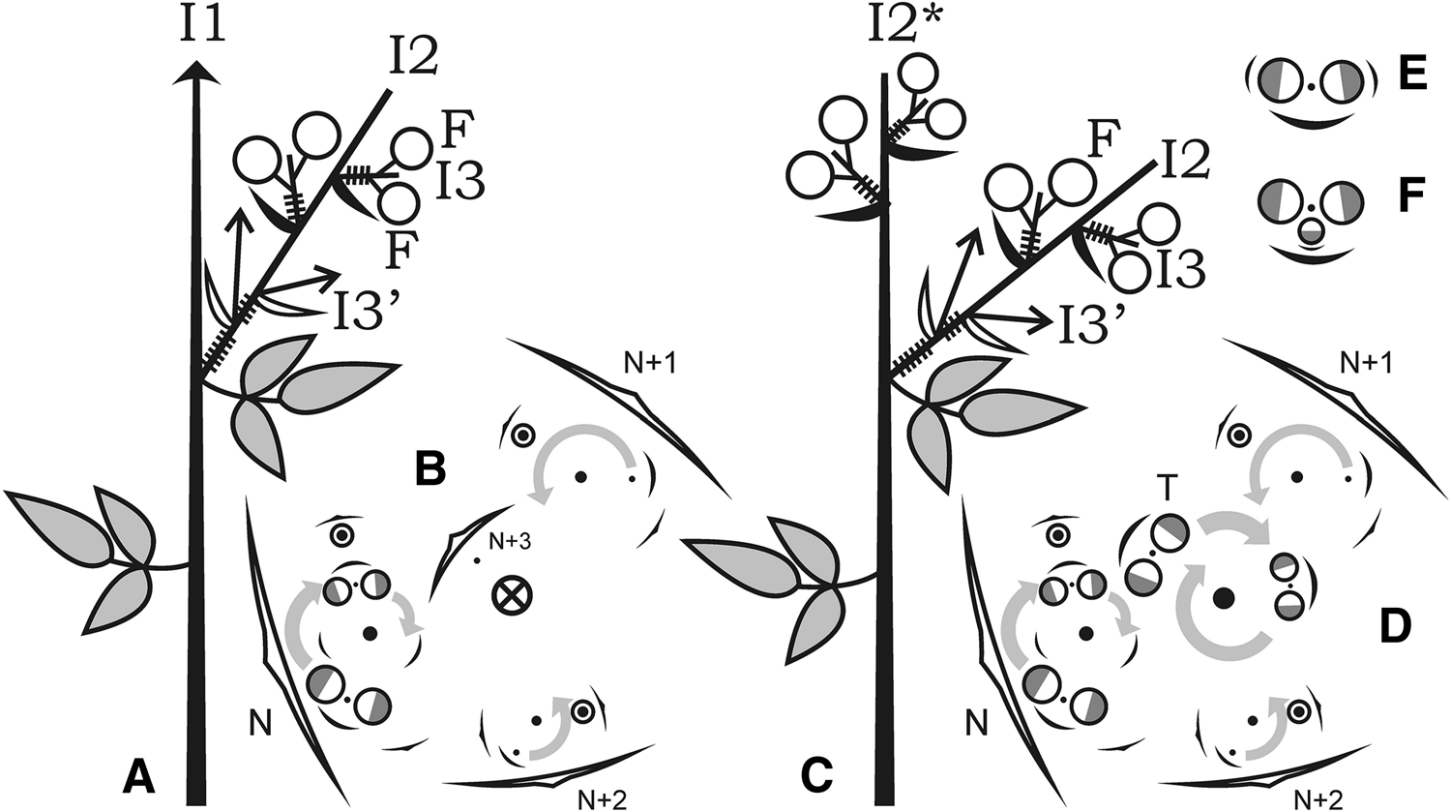
Schematic representations of inflorescence morphology in pigeonpea. *A*, *B*—IDT plant: scheme (*A*) and diagram (*B*). *Arrow* (*crossed circle on B*) continuously proliferating axis, *hatched* shortened internodes, *black arc* bract, *white arc* (*black acute arc on B*) prophyll, *white acute arc* frondose leaf, *half-grey circle* flower (*grey half* abaxial side), *black circle* axis with limited growth, *circle with dot* dormant axillary bud, *grey arrows* order of flowering, *N* node of flowering initiation, *T* terminal inflorescence. *C*, *D*—DT plant. *E*—axillary complex in line ICPL 87091. *F*—axillary complex in DT plants

**Fig. 2:**
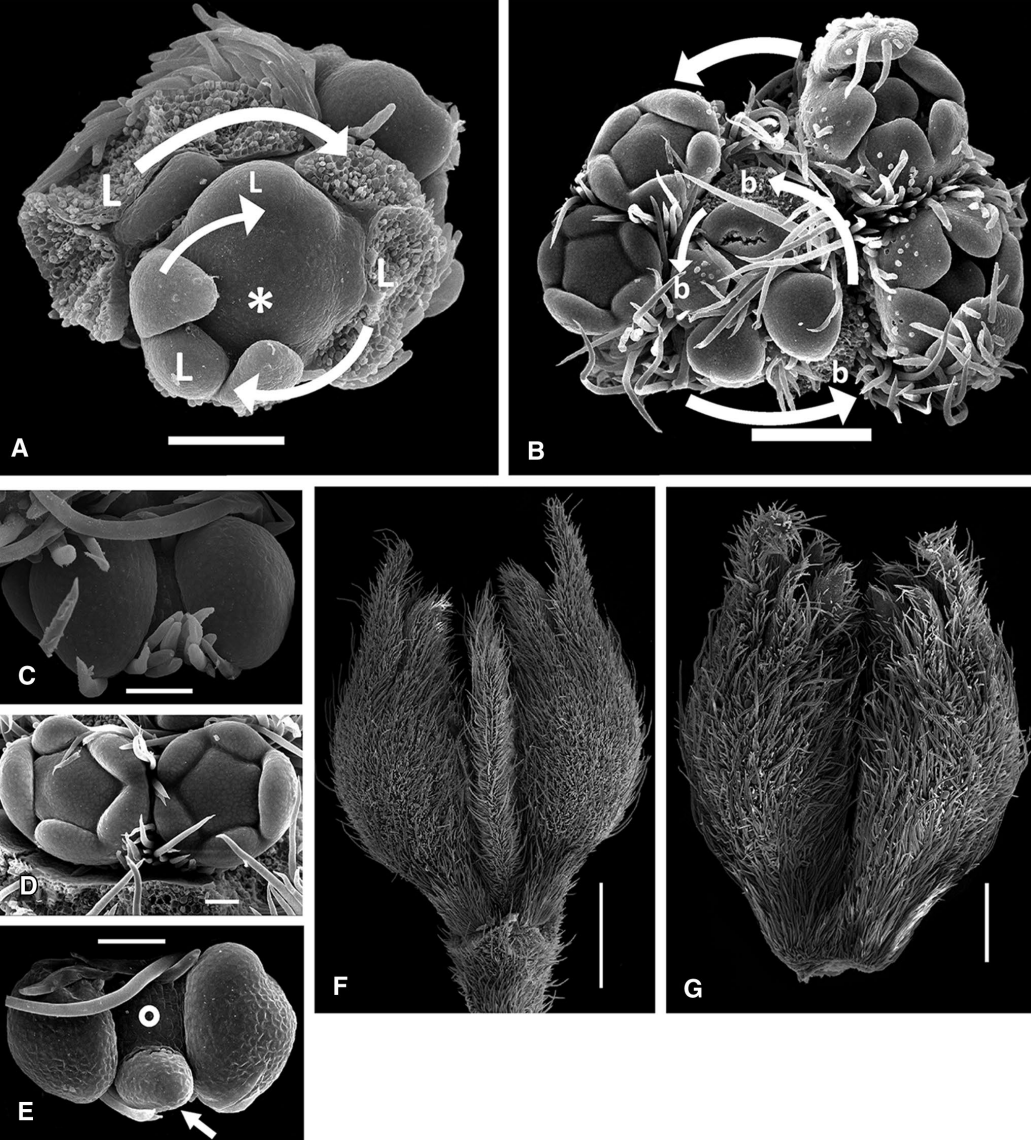
Apices of IDT (**a**, **c**, **d**, **g**) and DT (**b**, **e**, **f**) pigeonpea plants (SEM microphotographs). **a** Indeterminate vegetative apex (*asterisk*) of DT plant surrounded with primordia of frondose leaves (*L*) which comprise *right* spiral. *Arrow* order of initiation. **b** Apex of DT plant with pairs of flowers arising in axils of bracts (*b*) arranged in *left* spiral. **c** Flowers arising on I3 apex (seen between floral primordia). **d** Pair of flowers on later stage. **e** Flower pair of DT plant; I3 apex (*dot*) also produces phyllome (*arrow*). **f** Pair of flowers of DT plant with pin-like phyllome protruding between them. **g** Flower pair (IDT plant), preanthetic stage. *Scale bars* 50 μm (**c**–**e**), 100 μm (**a**), 200 μm (**b**), 500 μm (**g**), 1 mm (**f**)

IDT genotypes have been characterized with cryptocotylar hypogeal germination, i.e., cotyledons remain in a seed coat not emerging from ground. The first aerial node bears two opposed single-leaflet leaves. The second node bears two opposed leaves of either the same morphology as the first, or one of them was of single leaflet while another trifoliate, as on higher nodes. In few plants, a phyllotaxis was alternate, since the second node, but in this case, all leaves except for the first node were trifoliate. After transition to alternating phyllotaxis, stems of pigeonpea exhibit a handedness. Leaves were arranged in spirals, either right (clockwise) or left (counterclockwise) (Figs. 1, 2a). The angle between leaves is 137.5°. Although limited samples of each genotype were tested, ratio between right- and left-handed plants seems close to 1:1 (7:6 in ICPL 20325, *χ* ^2^ = 0.077, *p* < 0.05). After producing few leaves with vegetative buds in their axils, plants begin flowering. The first node which produces a lateral flower-bearing axis would further be designated as a node of flowering initiation (NFI). An average number of nodes preceding NFI vary in different genotypes (7–10 in ICPL 20325, 8–13 in ICPL 88039). In the case of DT pigeonpea genotypes, peculiarities of germination and vegetative growth were found similar to IDT type. Ratio between right- and left-handed plants was also similar to 1:1 (13:11 in ICPL 20338, *χ* ^2^ = 0.167, *p* < 0.05; 9:11 in ICPL 87091, *χ* ^2^ = 0.200, *p* < 0.05). The number of sterile nodes preceding NFI was variable depending on genotype and a certain plant (12–17 in ICPL 87091, 5–7 in ICPL 20338).

#### Inflorescence structure

IDT pigeonpea genotypes beginning from NFI, every leaf on the main (first order, I1) stem subtends an axillary (second order, or partial) racemose inflorescence (I2, Fig. 1a). A phyllotaxis of floral zone of the main shoot follows the same pattern as a preceding vegetative portion. The I1 stem proliferates unlimitedly. Every axillary partial inflorescence begins with two scale-like persistent prophylls which remain at its base, in an axil of frondose leaf (Fig. 1a). Each prophyll subtends a bud. In *C. cajan* grown in a glasshouse, these buds usually remain dormant, but one of them was developed more fully than another. This asymmetry indicated that prophylls were arranged in alternating (distichous) way rather than opposed. In *C. cajanifolius* inflorescences, one or both buds in prophylls’ axils usually unfold producing the third-order racemose inflorescences (I3′, Fig. 1a) similar to one which bears prophylls. A similar phenomenon was observed in *C. cajan* grown in a field. After producing two prophylls, a partial inflorescence begins initiation of bracts. Bracts were of oblonged ovate shape, with acute tip, densely pubescent. Arrangement (phyllotaxis) of bracts on axis was also spiral, with divergence angle which seems similar to one of the main axis. In many cases, torsion angles on the first- and second-order axes were converse. For example, if the main stem torsion was right, most (usually not all) of the axillary inflorescences would be left. In some plants, this right–left conversion between the first- and second-order axes was perfect, but in most cases, it had exclusions. The third-order racemose inflorescences (I3′), if unfold, often had a torsion converse compared with the second-order inflorescences on which they develop. This regularity was also not strictly followed. On every partial inflorescence, the first bract was initiated closer to the prophyll which subtends more developed bud. This pattern was obeyed rigorously, so chirality of a partial inflorescence can be predicted basing on position of prophylls (Fig. 1b). Every bract subtends a pair of flowers (Fig. 1b) which were initiated at apex as a loaf-shaped meristem primordium (Fig. 2b) which later splits into two floral primordia (Fig. 2b, c). A remaining meristem between them comprises an apex of shortened third-order axis (I3) which stops its proliferation after production of two flowers (Fig. 2d). These flowers seem to develop somewhat asynchronously, i.e., are initiated in successive mode. This phenomenon can also be seen at flowering stage when two flowers in pair open one after another, not simultaneously. Flowers themselves were not subtended by any discernible bracts nor bear bracteoles. Among all examined genotypes, we found only ICPL 87091, in which individual flowers were preceded by small-scaly abscising bracts (Fig. 1e). Heritability of this feature is yet to be studied. Flowers were positioned with their adaxial sides to each other (Fig. 2d, g). The number of nodes on I2 which produce bracts and I3 varies along the shoot together with length of sterile portion of I2. Length of the latter decreases in acropetal direction, as does number of flowers. However, in both field and greenhouse conditions, flowers of distal portions of I2 senesce before opening, so a potential productivity were never achieved and can hardly be estimated precisely.

**Fig. 3:**
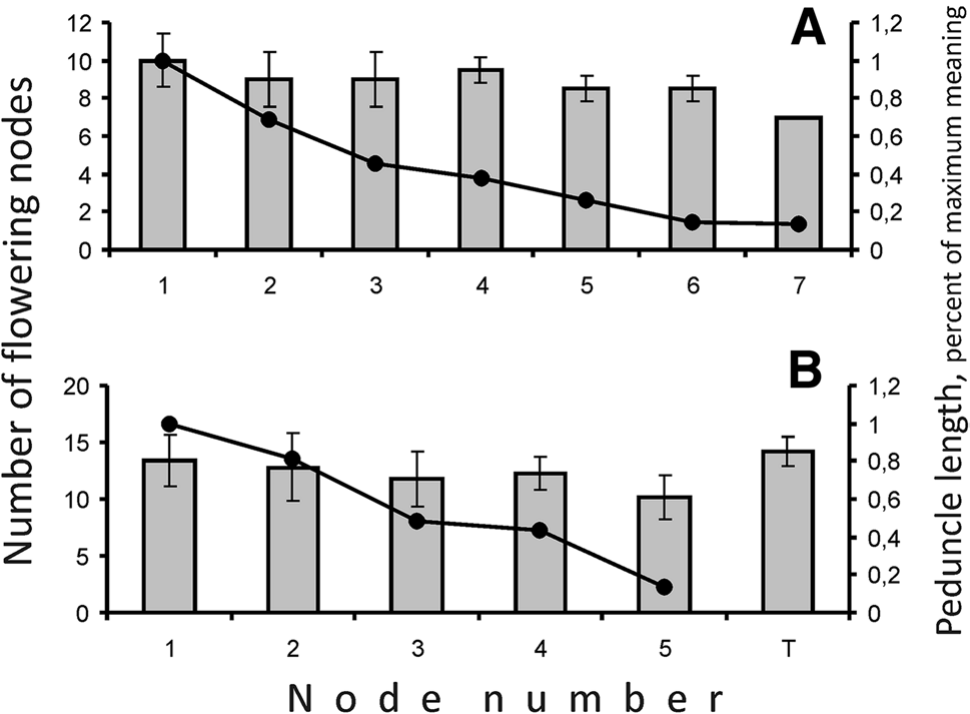
Variation in axillary inflorescences’ structure in IDT (**a**) and DT (**b**) plants depending on node number. *Number* of flowering nodes is depicted as polygon, relative peduncle length as *bars* (average ± standard deviation). *1* Node of flowering initiation, *T* terminal inflorescence

In DT pigeonpea genotypes as compared with indeterminate genotypes, plants exhibit transition of I1 to production of bracts instead of frondose leaves. These bracts subtend pairs of flowers, like on I3 (Figs. 1c, 2b). A phyllotaxis of terminal inflorescence continues one of the preceding zone of axillary inflorescences (Fig. 1d). In studied DT genotypes, production of additional organs on I3 was observed. Some of I3 apices also produced the third flower or bract-like phyllome (Fig. 2e, f). Such morphogenesis pattern in IDT plants was not detected, so we suppose that these features were associated. The number of flowering nodes in terminal inflorescence always exceeds one in previous axillary pseudoraceme and seems similar to the pseudoraceme which arises in NFI (Fig. 3b). In IDT plants, every successive node produces fewer flowers than preceding one (Fig. 3a).

### Inheritance of growth habit

A complete dominance of IDT was observed in F_1_s generated by crossing parental genotypes, namely, ICP 5529 (IDT) with ICP 11605 (DT). In F_2_ generation, a total of 202 individuals were phenotyped for IDT and DT growth habits. The F_2_ population segregated in 3 IDT: 1 DT ratio with probability value of 0.68 suggesting that IDT is controlled by a single dominant gene (Electronic supplementary material Table 1). In F_3_ generation, pooled analysis of 1597 F_3_ plants from randomly chosen 126 F_2_s, 855 F_3_ plants from 48 F_2_s segregated for IDT/DT, while 742 F_3_ from 78 F_2_s did not segregate. This segregation pattern fit well to the expected ratio of 1 segregating: 1 non-segregating (*p* = 0.30) (Electronic supplementary material Tables 2 and 3). The variation observed within 48 segregating progenies indicated the presence of a single gene with its dominant allele causing IDT, and segregated in 3 IDT:1 DT ratio (Electronic supplementary material Table 4).

### Sequence data and SNPs’ discovery

Parental genotypes (ICP 5529 × ICP 11605) together with 179 from a total of 188 F_2_s were sequenced, and a total of 305.53 million reads containing 30.85 Gb data were generated. DNA from 9 F_2_s could not be used for library preparation due to low concentration and quality. From the above-mentioned sequencing data in parental lines, a total of 5.37 million reads containing 0.54 Gb for ICP 5529 and 1.60 million reads containing 0.16 Gb for ICP 11605 were generated. In 179 F_2_s, the number of reads generated varied from 0.40 to 5.25 millions with an average of 1.66 million per F_2_ line (Electronic supplementary material Table 5). As a result, generated sequencing data on 179 F_2_ lines were used for SNPs identification and subsequently parsed to remove heterozygous SNPs in parental genotypes. Following stringent filtering criterion, a total of 12,654 SNPs were identified across 179 F_2_s.

### *CcTFL1*-derived Indel marker and segregation

To validate our hypothesis of association of *CcTFL1* gene to the growth habit in pigeonpea and to develop a user-friendly marker, we took advantage of available *CcTFL1* gene sequence from Mir et al. (2014) and re-sequencing data on determinate and indeterminate genotypes (Kumar et al. 2016). As a first instance, *CcTFL1* gene sequence was aligned across parental genotypes (ICP 5529 and ICP 11605) of F_2_ mapping population and reference pigeonpea genome. Sequence alignment has detected five potential variations in *CcTFL* gene region (Electronic supplementary material Table 6). Out of five sequence variations detected between ICP 5529 and ICP 11605, four were heterozygous in either of the parent. A 10 bp deletion has been identified on CcLG03pseudomolecule in DT genotype ICP 11605 while comparing with IDT genotype ICP 5529. Annotation of identified Indel has shown its active role in creating a frame shift mutation. Further flanking sequences of Indel region were used for primer designing. The primer pair designed for Indel region [CcLG03_ 20698771_F (CAT GGC CAT TGT AGA CTT GCT (21 bp) and CcLG03_ 20698771_R (TCA CAG CAG GAT CAT CGA GT (20 bp)] has been used to genotype F_2_ population derived from cross ICP 5529 × ICP 11605 (Electronic supplementary material Figure 1). The *CcTFL1* fragment containing 10 bp Indel segregated in the F_2_ population suggesting that *CcTFL1* is a candidate gene for growth habit and this marker was termed S3_20698771.

Marker S3_20698771 differentiated all 188 F_2_ plants into 148 IDT plants and 40 DT plants on agarose gel. All determinate plants were homozygous for the marker S3_20698771. Among indeterminate F_2_ plants, 107 were heterozygous and 41 were homozygous for the marker S3_20698771. A goodness-of-fit test for a 1 determinate (homozygous):2 indeterminate (heterozygous):1 indeterminate (homozygous) provided a *χ* ^2^ value of 3.6 at probability value of 0.16 (Electronic supplementary material Table 7).

### SNPs-based genetic map

**Fig. 4:**
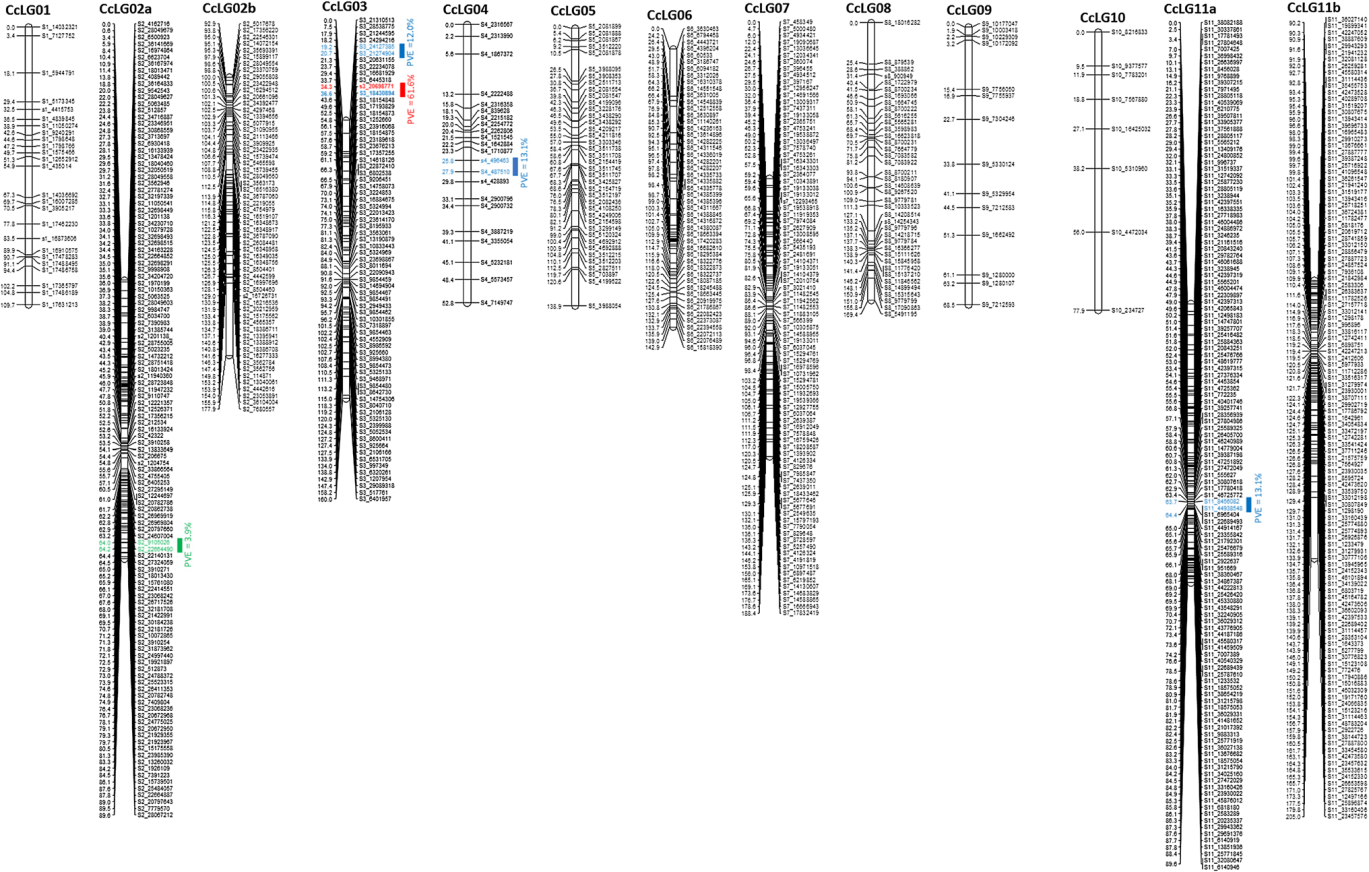
Gentic map and QTLs for the ICP 5529 × CP 11605 mapping population. *Bars* indicate QTL positions: *Red bar* is a major QTL with one of the two flanking markers being CcLG03_20698771 deletion marker derived from the *CcTFL1* gene (Mir et al. 2014) with the PVE of 61.6, *Blue bars* represents other major QTLs and *Green bar* is a minor QTL. All coloured markers are flanking QTL positions

From a total of 12654 SNPs identified, 2935 SNPs showed segregation in 1:2:1 ratio with a cut-off probability value 10^−9^ and retained for genetic mapping. Further marker genotyping data generated through *CcTFL1*-derived Indel marker (S3_20698771) on F_2_ population was also added in SNP data set for constructing genetic map. As segregation distortion from the expected ratio was on higher side in the F_2_ population, a subgroup of 714 markers with a cut-off probability value ≥0.05 was created and considered as anchor markers for initiating genetic map construction. As a result, a total of 140 markers with probability value ≥0.05 could be mapped in framework genetic map. Furthermore, 647 markers with probability value <0.05 ≥ 10^−9^ could be integrated into framework genetic map accounting a total of 787 markers in the genetic map with 1454 cM map length (Fig. 4). The highest number of markers was mapped on CcLG11 (249), while the lowest number of markers was mapped to CcLG10 (8). Length of linkage groups varied from 40.5 cM (CcLG10) to 205 cM (CcLG11). Overall, map had 0.54 markers per cM on average (Table 1).

**Table 1:** Details on intra-specific genetic map developed for F_2_ population (ICP 5529 × ICP 11605)

CcLGs	Number polymorphic SNPs			Framework map*		Final genetic map			
	Total	*p* ≥ 1.0E−9	*p* ≥ 0.05	Number of markers	Length	*p* < 0.05 ≥ 1.0E−9	Total	Length (cM)	Interval (per marker)
01	956	179	36	4	13.4	21	25	109.7	4.386
02	1811	452	105	35	110.8	144	179	177.9	0.994
03	1183	195	53	15	109.5	58	73	160	2.191
04	671	165	69	15	36.4	7	22	52.8	2.399
05	437	93	21	3	2	36	39	138.9	3.562
06	1183	292	58	28	74.5	21	49	142.9	2.917
07	915	246	53	9	51.9	81	90	188.4	2.094
08	909	186	67	7	30.3	32	39	169.5	4.346
09	583	129	38	8	47.2	6	14	68.5	4.892
10	1272	322	84	3	25.4	5	8	40.5	5.068
11	2742	676	130	13	82.7	236	249	205.0	0.823
Total	12662	2935	714	140	584.2	647	787	1454.1	1.847

* Constructed with markers segregating in a 1:2:1 expected F_2_ ratio at *χ*2 prob. of 0.05

### QTLs for growth habit

**Fig. 5:**
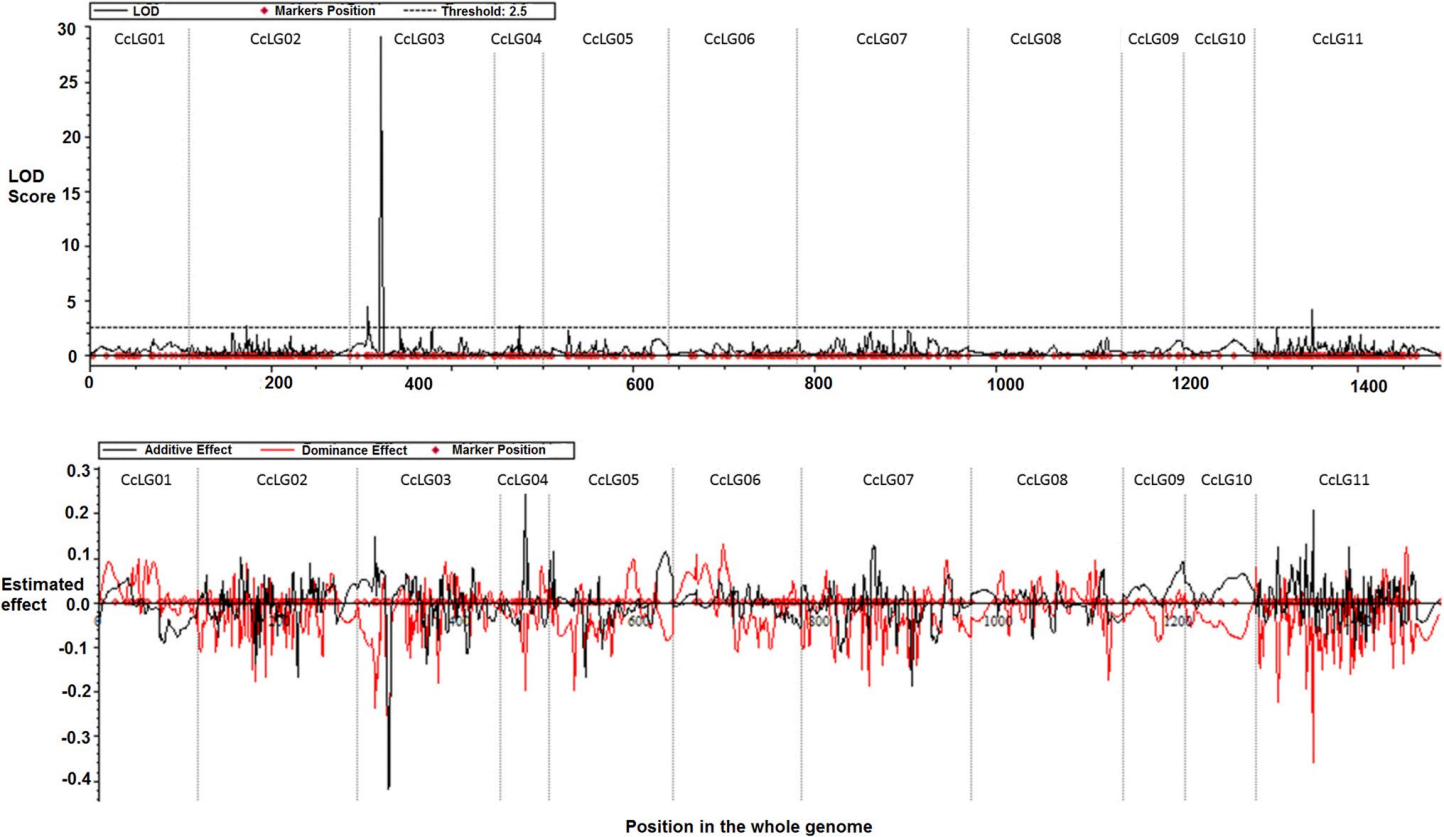
Genome-wide QTL positions for growth habit and their effects in ICP 5529 × ICP 11605 mapping population. QTLs were detected by inclusive composite interval mapping (ICIM) in the QTLIcim software v4.0

QTL mapping for growth habit was performed using trait phenotypic data together with marker genotyping data on the F_2_ mapping population following inclusive composite interval mapping (ICIM). Marker genotyping data included 786 SNPs and one *CcTFL1-*derived Indel marker. ICIM identified a total of five QTLs for growth habit in pigeonpea. Out of these identified five QTLs, four QTLs were considered as major effect QTLs (explaining more than 10% of phenotypic variation). Whereas one QTL identified on CcLG02 was considered as minor effect QTL explaining 3.9% of phenotypic variation. Among major effect QTLs, two QTLs were located on CcLG03 and one QTL each on CcLG04 and CcLG11. The QTL flanked by S3_24127385 and S3_21274904 markers on CcLG03 explained 12% of phenotypic variation at 4.4 LOD value. Another QTL flanked by S3_20698771 and S3_18430894 markers on CcLG03 explained 61.6% of phenotypic variation at 29.1 LOD value. It is interesting to note that marker S3_20698771 has been derived from an Indel identified in genic region of *CcTFL1* and explaining almost two-third of total of phenotypic variation explaining growth habit in pigeonpea. QTL flanked by S4_496463 and S4_487510 markers on CcLG04 explained 13.1% of phenotypic variation at 2.6 LOD value. The fourth major QTL was on CcLG11 flanked by S11_8456082 and S11_44938548 markers explained 14% of phenotypic variation at 4.1 LOD value (Table 2; Fig. 5).

**Table 2:** Genome-wide QTL positions and effects in ICP 5529 × ICP 11605 F_2_ mapping population detected by inclusive composite interval mapping (ICIM) for growth habit

CcLGs	Position (cM)	Left flanking marker	Right flanking marker	LOD value	PVE (%)
02	64	S2_9105026	S2_22664490	2.7	3.9
03	20	S3_24127385	S3_21274904	4.4	12.0
03	35	S3_20698771	S3_18430894	29.1	61.6
04	27	S4_496463	S4_487510	2.6	13.1
11	64	S11_8456082	S11_44938548	4.1	14.0

### Validation of *CcTFL1*-derived Indel marker

To validate association of *CcTFL1*-derived Indel marker (S3_20698771) with growth habit in pigeonpea, a set of 262 genotypes was used. This set comprised of 16 DT, 10 SDT, and 236 IDT genotypes. Primer pairs used to amplify marker S3_20698771, a single amplification product of 200 bp was obtained in all IDT and SDT genotypes. In all the 16 DT genotypes, a single amplification product of 190 bp was obtained using similar primer pairs mentioned above (Electronic supplementary material Figure 2).

## Discussion

The apical meristem of shoot is a set of stem cells persistent at the shoot axis. It proliferates and generates leaves with axillary organs, either vegetative shoots or flowers (Bowman et al. 1993). The fate of apical meristem was studied thoroughly in *Arabidopsis* and depends on generally two sets of genes: first set of genes such as *LEAFY* (*LFY*), *APETALA1* (*AP1*) and *CAULIFLOWER* (*CAL*) converts meristem into flowers rather than shoots or leaves (Bowman et al. 1993; Gustafson et al. 1994; Kempin et al. 1995; Mandel and Yanofsky 1995). Second set of genes such as *TERMINAL FLOWER1* (*TFL1*) represses flower generation from apical meristem and gives rise to indeterminate growth habit (Bradley et al. 1996). *TFL1* gene also contributes to flowering time, influencing phase expansion and inflorescence formation in *Arabidopsis* (Bradley et al. 1997; Ruiz-García et al. 1997). *TFL1* leads down regulation of *LFY* and *AP1* genes, hence prevent their expression in apical meristem. Mutation in *TFL1* converts apical meristem to terminal flower and gives rise to determinate type of growth habit. The vegetative and reproductive durations have been significantly shortened in *TFL1* mutant plants (Ahn et al. 2006). Due to significance of growth habit trait, a number of attempts have been made to detect the homologous genes of *TFL1* and understand its mechanism in different plant species such as brassica, pea, grapevine, lotus, tomato, tobacco, apple, cotton, cucumber, pepper, soybean, common bean, pigeonpea, etc. To characterize growth habit trait in pigeonpea, a comprehensive approach has been followed in the present study.

Pigeonpea is a member of Phaseoleae tribe which is one of the most diverse in legume family. In pigeonpea, bracts on I2 subtend extremely shortened I3 axes which usually bear two flowers, the latter developing in alternate way. Such inflorescence type is called pseudoraceme (Tucker, 1987). Stem apical meristem (SAM) of I3 was seen only during flower initiation (Fig. 2c) and indistinguishable by anthesis. Flowers were initiated in axils of prophylls which arise in transversal position and usually remain cryptic themselves. We found a single genotype in which they develop as bracts; this trait may be heritable. Pigeonpea plants have spiral phyllotaxis. A phyllotaxis torsion of any individual plant may be either right or left. In *Arabidopsis*, a suite of mutants which cause development of exclusively right- or left-handed plants was isolated (Hashimoto 2002). In pigeonpea, we recorded both types in all examined genotypes, and ratio between plants with different torsion corresponded significantly to 1:1. Although Bahadur and Rao (1981) after analyzing larger sample reported that ratio between different chirality types significantly deviates from 1:1, we propose that this feature has no genetic determination in our studied material. Otherwise, one can hardly imagine any ratio among isogenic plants. A phyllotaxis of terminal inflorescence (I2*, Fig. 1c) continues one of preceding I1 zone. This continuation is evident from both divergence angle and torsion, either right or left. A similar pattern was recorded in DT forms of pea and broad bean (Sinjushin 2013), where the first bract of terminal raceme is usually placed exactly, where the frondose leaf of preceding shoot should develop. A terminal inflorescence (I2*) was larger than preceding lateral ones (I2) (Fig. 3b), as it includes more nodes with axillary I3s. We observed production of additional structures on I3 of DT plants (Fig. 2e, f). These structures may have dorsoventral habit and seemingly comprise phyllomes (bracts). Sometimes, the third flower with subtending bract develops in median plane (Fig. 1f). These structures do not seem truly terminal on I3: the I2 apex is visible (Fig. 2e). In *Arabidopsis*, gene *TFL1* controls both inflorescence determinacy and flowering time, as mutants *tfl1* begin flowering significantly earlier than wild-type plants (Hanano and Goto 2011). As it had been shown in pea, gene *TFL1* has three orthologs (Foucher et al. 2003). One of them, *PsTFL1a*, corresponds to gene *DETERMINATE* (*DET*) which prevents SAM from production of apical raceme. Another, *PsTFL1c*, comprises gene *LATE FLOWERING* (*LF*) which defines flowering time: NFI in *lf* mutants has lower number than in *LF* plants. Thus, two functions present in *Arabidopsis* become distributed between two *TFL1* orthologs in pea.

### *Dt*_*1*_ locus responsible for indeterminacy in pigonpea

Limited information is available to understand the genetic control of growth habit in pigeonpea (Shaw 1936; Reddy and Rao 1974; Saxena and Sharma 1990; Gupta and Kapoor 1991; Gumber and Singh 1997; Sapkal et al. 2015). Through the above-mentioned inheritance studies, it has been proposed that growth habit in pigeonpea is controlled by two dominant genes (*Dt*_*1*_ and *Dt*_*2*_) with inhibitory interaction. *Dt*_*1*_ is responsible for IDT phenotype and suppresses the expression of *Dt*_*2*_. IDT phenotype can be produced when a plant has either *Dt*_*1_*_ *Dt*_*2_*_ or *Dt*_*1_*_ *dt*_*2*_ *dt*_*2*_ combinations. SDT phenotype can be produced when a plant has *dt*_*1*_ *dt*_*1*_ *Dt*_*2_*_ combination. The DT phenotype can only be produced when both the genes are present in recessive and homozygous condition, i.e., *dt*_*1*_ *dt*_*1*_ *dt*_*2*_ *dt*_*2*_. In the present study, a segregating population was developed using IDT and DT parental genotypes. The segregation observed (3 IDT: 1 DT) in the present study supports the hypothesis of single dominant gene controlling IDT growth habit in pigeonpea. Similar observations have been made in soybean (Bernard 1972) and broad bean (Brimo 1983). SDT phenotype was also investigated in pigeonpea and appeared recessive with response to IDT (Gupta and Kapoor 1991). Studies conducted in pigeonpea with SDT × IDT cross uncovered dominant epistasis (i.e., 12 IDT:3 SDT:1 DT individuals in F_2_ progeny) (Gupta and Kapoor 1991; Gumber and Singh 1997). This ratio has also been found in soybean (Bernard 1972), chickpea (Hegde 2011), and tomato (Elkind et al. 1991). In pigeonpea, chickpea, and tomato, SDT has been found recessive trait but dominant in soybean.

Comparisons of soybean with *Arabidopsis* have shown that *Dt1* was an ortholog of *TERMINAL FLOWER1* (*TFL1*) gene (Liu et al. 2010; Tian et al. 2010). *TFL1* also involved in the transition from indeterminate phenotype to determinate phenotype (Tian et al. 2010). Therefore, it is worthwhile to map the *Dt1* locus in pigeonpea and assess its association with *CcTFL1* a homolog of *TFL1*. It is important to mention that in our previous study, *CcTFL1* was identified (Mir et al. 2014); however, its association with *Dt1* locus was unknown.

The genetic map developed in the present study covered all the 11 linkage groups of pigeonpea. Total length of this genetic map (1454 cM) was on higher side as compared to previously developed few simple sequence repeats (SSRs) and F_2_ population-based intra-specific genetic maps in pigeonpea (Bohra et al. 2012). Whereas, the genetic map developed on F_2_ population derived from Pusa Dwarf’ and ‘HDM04-1’ using 296 genic SNP and SSR markers covered 1520.22 cM (Kumawat et al. 2012). In terms of number of markers mapped in any intra-specific population, the present genetic map has been the most saturated map in pigeonpea as it harbors a total of 787 markers. Few linkage groups in the present genetic map possess a highly dense coverage, while other contained gaps. Inflated length in the present genetic map may be due to segregation distortion observed for a number of SNPs. It is important to mention that GBS has been used to generate SNP genotyping data on the F_2_ population. GBS seems to be the best option to generate thousands of SNPs in short period of time in any given population. However, reduced representation of genome, problems of missing data, and false-negative genotyping calls especially in detecting heterozygotes in F_2_ population might have added to the segregation distortion of SNPs generated in the present study. The segregation distortion in pigeonpea F_2_ populations was observed not only for GBS-based SNPs data, but also for allele-specific polymerase chain reaction (KASPar)-based SNPs data (Saxena et al. 2012) and microsatellite markers (Bohra et al. 2012). The reasons for this segregation distortion could be associated with inherent nature of markers or limitations in genotyping strategy as mentioned above and selection of adaptive genomic regions in breeding cycle of the mapping population development. However, segregation distortion in markers did not exclude them from being potentially informative. In fact, it has been identified that the use of distorted markers could increase the mapping saturation and statistical analysis power for QTL detection (Shizhong 2008a, b). Therefore, in the present study, distorted markers were also included into the linkage analysis.

Mapping data together with DT/IDT phenotyping data on F_2_ mapping population provided candidate molecular markers and QTLs for growth habit in pigeonpea. The four QTLs detected for growth habit contributed more than 10% of phenotypic variation. These four QTLs have shown contrasting parental effects (three QTLs with positive and one QTL with negative additive effects) including two major QTLs had close map positions on CcLG03; the contrasting parental effects suggested that different QTLs are involved in shaping growth habit in pigeonpea. One QTL from the above-mentioned four QTLs flanked by S3_20698771 and S3_18430894 markers contributing almost two-third of phenotypic variation. This region can be considered as locus for *Dt*_*1*_ in pigeonpea. One marker S3_20698771 flanking this QTL has been derived from Indel identified in *CcTFL1* region. Therefore, location of *CcTFL1* in the QTL region further enhances our confidence in defining this region as *Dt*_*1*_. These results are in continuation of our previous findings, where we have identified *CcTFL1* gene as a potential candidate gene associated with growth habit in pigonpea through sequence polymorphism, candidate gene mapping, and comparative genomics analysis (Mir et al. 2014). Growth habit traits have also been mapped in some other crops species such as soybean, pea, and common bean (Foucher et al. 2003; Kwak et al. 2008; Tian et al. 2010). However, the marker developed by Mir et al. (2014) based on SNP identified (A/T) in *CcTFL1* gene was not user friendly; therefore, in the present study, we have analyzed *CcTFL1* gene sequence in parental lines of mapping population and detected a 10 bp deletion in DT parent. Functional annotation of identified Indel suggested a frameshift mutation in DT genotype. Furthermore, in comparison with other marker systems, Indels have a number of advantages such as multi-allelic and co-dominant, ease in marker conversion, and most importantly amenable to low-cost genotyping. Indels have been found responsible in altering the gene functions (Jiang et al. 2012). This newly developed Indel-derived marker co-segregated with *Dt1* locus in mapping population. Subsequently, this marker has been validated on a set of 262 pigeonpea lines.

The present study in conjunction with Mir et al. (2014) proves that *CcTFL1* is a likely candidate gene for growth habit in pigeonpea. The Indel-derived marker based on *CcTFL1* will be useful in marker-assisted breeding programs and would allow early generation selection efficiency in crossing programs to select both DT and IDT lines depending on the objective. In the near future, a system level approach to elucidate the gene networks that modulate shoot apical meristem and inflorescence architecture will be required to understand the biological mechanism controlling growth habit in pigeonpea.

#### Author contribution statement

RKS and RKV conceived, designed, and coordinated the experiments. JO, CVSK, and KBS performed the field experimentations. RKS and JO performed the genotyping. RKS, JO, KBS, and RKV analyzed the data. AS performed the SEM analysis. RKS, JO, AS, and RKV interpreted results. RKS, AS, KBS, and RKV wrote the paper.

## Electronic supplementary material

Below is the link to the electronic supplementary material.

Supplementary material 1 (DOCX 36 kb)

Supplementary material 2 (PPTX 229 kb)

Supplementary material 3 (PPTX 653 kb)

Supplementary material 4 (XLSX 15 kb)
