# Supplementary material for: Characterization and mapping of Dt1 locus which co-segregates with CcTFL1 for growth habit in pigeonpea
Source: Theor Appl Genet. 2017 May 24;130(9):1773–84. doi: 10.1007/s00122-017-2924-2 (PMC5565653; doi:10.1007/s00122-017-2924-2)
Supplement: Supplementary file 1 — Supplementary material 1 (DOCX 36 kb) [file 122_2017_2924_MOESM1_ESM.docx]

**Electronic supplementary material (ESM):**

ESM Table 1. Chi square goodness-of-fit to expected 3:1 F_2_ phenotypic segregation ratio for growth habit

| **Total** | **No. of observed IDT F_2_s** | **No. of observed DT F_2_s** | **No. of expected IDT F_2_s** | **No. of expected DT F_2_s** | **χ^2^ value** | **Probability (p value)** |
| --- | --- | --- | --- | --- | --- | --- |
| 202 | 154 | 48 | 151.5 | 50.5 | 0.165 | 0.68 |

IDT = indeterminate, DT = determinate

ESM Table 2. Non-segregating indeterminate (IDT) and determinate (DT) F_2_ plants in F_3_ generation

| **F_2_ plant ID** | | **Observed growth habit in F_2_s** | | **Total number of F_3_ plants** | **Observed-IDT F_3_ plants** | **Observed-DT F_3_ plants** |
| --- | --- | --- | --- | --- | --- | --- |
| Indeterminate | |  | |  |  |  |
| 2 | | IDT | | 24 | 24 | 0 |
| 5 | | IDT | | 6 | 6 | 0 |
| 7 | | IDT | | 17 | 17 | 0 |
| 8 | | IDT | | 14 | 14 | 0 |
| 20 | | IDT | | 23 | 23 | 0 |
| 24 | | IDT | | 3 | 3 | 0 |
| 26 | | IDT | | 12 | 12 | 0 |
| 29 | | IDT | | 15 | 15 | 0 |
| 30 | | IDT | | 18 | 18 | 0 |
| 32 | | IDT | | 2 | 2 | 0 |
| 36 | | IDT | | 15 | 15 | 0 |
| 37 | | IDT | | 7 | 7 | 0 |
| 40 | | IDT | | 15 | 15 | 0 |
| 41 | | IDT | | 14 | 14 | 0 |
| 45 | | IDT | | 6 | 6 | 0 |
| 84 | | IDT | | 23 | 23 | 0 |
| 86 | | IDT | | 23 | 23 | 0 |
| 88 | | IDT | | 22 | 22 | 0 |
| 98 | | IDT | | 22 | 22 | 0 |
| 120 | | IDT | | 20 | 20 | 0 |
| 123 | | IDT | | 16 | 16 | 0 |
| 124 | | IDT | | 5 | 5 | 0 |
| 128 | | IDT | | 20 | 20 | 0 |
| 133 | | IDT | | 17 | 17 | 0 |
| 138 | | IDT | | 17 | 17 | 0 |
| 145 | | IDT | | 18 | 18 | 0 |
| 148 | | IDT | | 20 | 20 | 0 |
| 151 | | IDT | | 4 | 4 | 0 |
| 155 | | IDT | | 17 | 17 | 0 |
| 158 | | IDT | | 5 | 5 | 0 |
| 161 | | IDT | | 5 | 5 | 0 |
| 162 | | IDT | | 5 | 5 | 0 |
| 166 | | IDT | | 1 | 1 | 0 |
| 171 | | IDT | | 2 | 2 | 0 |
| 178 | | IDT | | 3 | 3 | 0 |
| 179 | | IDT | | 1 | 1 | 0 |
| 180 | | IDT | | 6 | 6 | 0 |
| 183 | | IDT | | 1 | 1 | 0 |
| 185 | | IDT | | 3 | 3 | 0 |
| 190 | | IDT | | 4 | 4 | 0 |
| 192 | | IDT | | 5 | 5 | 0 |
| 193 | | IDT | | 1 | 1 | 0 |
| 195 | | IDT | | 8 | 8 | 0 |
| 197 | | IDT | | 1 | 1 | 0 |
| 198 | | IDT | | 1 | 1 | 0 |
| 200 | | IDT | | 1 | 1 | 0 |
| 202 | | IDT | | 1 | 1 | 0 |
| 205 | | IDT | | 7 | 7 | 0 |
| 206 | | IDT | | 2 | 2 | 0 |
| 207 | | IDT | | 6 | 6 | 0 |
| 208 | | IDT | | 3 | 3 | 0 |
| 211 | | IDT | | 2 | 2 | 0 |
| 213 | | IDT | | 3 | 3 | 0 |
| 214 | | IDT | | 1 | 1 | 0 |
| 216 | | IDT | | 2 | 2 | 0 |
| 218 | | IDT | | 3 | 3 | 0 |
| 221 | | IDT | | 4 | 4 | 0 |
| 222 | | IDT | | 5 | 5 | 0 |
| 225 | | IDT | | 2 | 2 | 0 |
| 226 | | IDT | | 2 | 2 | 0 |
| 229 | | IDT | | 2 | 2 | 0 |
| Determinate |  | |  | |  |  |
| 1 | DT | | 13 | | 0 | 13 |
| 13 | DT | | 23 | | 0 | 23 |
| 21 | DT | | 19 | | 0 | 19 |
| 23 | DT | | 11 | | 0 | 11 |
| 31 | DT | | 20 | | 0 | 20 |
| 47 | DT | | 21 | | 0 | 21 |
| 107 | DT | | 5 | | 0 | 5 |
| 112 | DT | | 15 | | 0 | 15 |
| 118 | DT | | 23 | | 0 | 23 |
| 134 | DT | | 1 | | 0 | 1 |
| 136 | DT | | 21 | | 0 | 21 |
| 147 | DT | | 17 | | 0 | 17 |
| 150 | DT | | 4 | | 0 | 4 |
| 153 | DT | | 4 | | 0 | 4 |
| 157 | DT | | 2 | | 0 | 2 |
| 168 | DT | | 2 | | 0 | 2 |
| 201 | DT | | 8 | | 0 | 8 |

ESM Table 3. Pooled segregating vs non-segregating in F_3_ plants for expected ratio of 1:1

| **Total** | **Observed** | | **Test ratio** | **χ^2^ value** | **Probability (p value)** |
| --- | --- | --- | --- | --- | --- |
|  | **Segregating** | **Non-segregating** |  |  |  |
| 1597 | 855 | 742 | 1:1 | 1.08 | 0.30 |

ESM Table 4. Chi square goodness-of-fit to expected 3:1 heterozygous F_3_ plant segregation ratio for growth habit

| **Segregating F_2__IDTs** | | **Observed F_3_ segregation** | | | | | |
| --- | --- | --- | --- | --- | --- | --- | --- |
| **F_2_ Plant ID** | **Observed growth habit in F_2_s** | **Total number of F_3_ plants** | **IDT** | **DT** | **Test ratio** | **χ^2^ value** | **Probability (p value)** |
| 3 | IDT | 19 | 13 | 6 | 3:1 | 0.44 | 0.51 |
| 10 | IDT | 24 | 17 | 7 | 3:1 | 0.22 | 0.64 |
| 11 | IDT | 20 | 16 | 4 | 3:1 | 0.27 | 0.61 |
| 12 | IDT | 19 | 13 | 6 | 3:1 | 0.44 | 0.51 |
| 14 | IDT | 23 | 15 | 8 | 3:1 | 1.17 | 0.28 |
| 15 | IDT | 18 | 13 | 5 | 3:1 | 0.07 | 0.79 |
| 18 | IDT | 20 | 14 | 6 | 3:1 | 0.27 | 0.61 |
| 25 | IDT | 14 | 12 | 2 | 3:1 | 0.86 | 0.35 |
| 28 | IDT | 23 | 19 | 4 | 3:1 | 0.71 | 0.40 |
| 46 | IDT | 12 | 11 | 1 | 3:1 | 1.78 | 0.18 |
| 72 | IDT | 6 | 5 | 1 | 3:1 | 0.22 | 0.64 |
| 74 | IDT | 19 | 12 | 7 | 3:1 | 1.42 | 0.23 |
| 75 | IDT | 22 | 20 | 2 | 3:1 | 2.97 | 0.08 |
| 77 | IDT | 13 | 9 | 4 | 3:1 | 0.23 | 0.63 |
| 79 | IDT | 13 | 9 | 4 | 3:1 | 0.23 | 0.63 |
| 83 | IDT | 20 | 15 | 5 | 3:1 | 0.00 | 1.00 |
| 85 | IDT | 22 | 18 | 4 | 3:1 | 0.55 | 0.46 |
| 87 | IDT | 17 | 14 | 3 | 3:1 | 0.49 | 0.48 |
| 89 | IDT | 19 | 13 | 6 | 3:1 | 0.44 | 0.51 |
| 90 | IDT | 21 | 15 | 6 | 3:1 | 0.14 | 0.71 |
| 96 | IDT | 21 | 19 | 2 | 3:1 | 2.68 | 0.10 |
| 99 | IDT | 22 | 18 | 4 | 3:1 | 0.55 | 0.46 |
| 100 | IDT | 18 | 13 | 5 | 3:1 | 0.07 | 0.79 |
| 102 | IDT | 20 | 17 | 3 | 3:1 | 1.07 | 0.30 |
| 103 | IDT | 22 | 16 | 6 | 3:1 | 0.06 | 0.81 |
| 104 | IDT | 18 | 17 | 1 | 3:1 | 3.63 | 0.06 |
| 108 | IDT | 21 | 16 | 5 | 3:1 | 0.02 | 0.90 |
| 115 | IDT | 20 | 15 | 5 | 3:1 | 0.00 | 1.00 |
| 117 | IDT | 14 | 12 | 2 | 3:1 | 0.86 | 0.35 |
| 121 | IDT | 20 | 16 | 4 | 3:1 | 0.27 | 0.61 |
| 125 | IDT | 21 | 19 | 2 | 3:1 | 2.68 | 0.10 |
| 126 | IDT | 23 | 18 | 5 | 3:1 | 0.13 | 0.72 |
| 127 | IDT | 21 | 17 | 4 | 3:1 | 0.40 | 0.53 |
| 129 | IDT | 26 | 21 | 5 | 3:1 | 0.46 | 0.50 |
| 131 | IDT | 17 | 14 | 3 | 3:1 | 0.49 | 0.48 |
| 132 | IDT | 18 | 13 | 5 | 3:1 | 0.07 | 0.79 |
| 135 | IDT | 19 | 15 | 4 | 3:1 | 0.16 | 0.69 |
| 137 | IDT | 21 | 19 | 2 | 3:1 | 2.68 | 0.10 |
| 139 | IDT | 16 | 11 | 5 | 3:1 | 0.33 | 0.56 |
| 142 | IDT | 16 | 14 | 2 | 3:1 | 1.33 | 0.25 |
| 143 | IDT | 18 | 16 | 2 | 3:1 | 1.85 | 0.17 |
| 144 | IDT | 18 | 17 | 1 | 3:1 | 3.63 | 0.06 |
| 146 | IDT | 14 | 11 | 3 | 3:1 | 0.10 | 0.76 |
| 149 | IDT | 20 | 13 | 7 | 3:1 | 1.07 | 0.30 |
| 175 | IDT | 5 | 4 | 1 | 3:1 | 0.07 | 0.80 |
| 182 | IDT | 5 | 4 | 1 | 3:1 | 0.07 | 0.80 |
| 194 | IDT | 5 | 3 | 2 | 3:1 | 0.60 | 0.44 |
| 229 | IDT | 5 | 3 | 2 | 3:1 | 0.60 | 0.44 |

ESM Table 5. GBS data generated on parents and F_2_s

(Separate excel file)

ESM Table 6. Sequence variation identified in *CcTFL1* region on CcLG03

| **Position (bp)** | **Reference allele** | **Alternative allele** | **Allele in ICP 11605** | **Allele in ICP 5529** | **Effects** |
| --- | --- | --- | --- | --- | --- |
| 20697552 | C | CCT | C | C/CCT | EFF=FRAME_SHIFT(HIGH\|\|-/AG\|-164?\|\|Gene_C.cajan_10074\|\|\|C.cajan_10074\|4\|1) |
| 20697742 | A | T | N | T | EFF=INTRON(MODIFIER\|\|\|\|\|Gene_C.cajan_10074\|\|\|C.cajan_10074\|3\|1) |
| 20697902 | C | CCA | N | C/CCA | EFF=INTRON(MODIFIER\|\|\|\|\|Gene_C.cajan_10074\|\|\|C.cajan_10074\|2\|1) |
| 20698247 | T | A | W | T | EFF=INTRON(MODIFIER\|\|\|\|\|Gene_C.cajan_10074\|\|\|C.cajan_10074\|2\|1) |
| 20698771 | ATTTTTATGCTT | AT | AT | ATTTTTATGCTT | EFF=FRAME_SHIFT(HIGH\|\|-\|-24\|\|Gene_C.cajan_10074\|\|\|C.cajan_10074\|1\|1) |

ESM Table 7. Indel marker (S3_20698771) derived from *CcTFL1* segregation goodness-of-fit to 1:2:1 at p = 0.05 in F_2_s

| **Observed** | | | | **Expected** | | | **((O-E)^2)/E** | | |  |  |
| --- | --- | --- | --- | --- | --- | --- | --- | --- | --- | --- | --- |
| Total | a | h | b | a | h | b | a | h | b | χ2 | P |
| 188 | 41 | 107 | 40 | 47 | 94 | 47 | 0.765957 | 1.797872 | 1.042553 | 3.606383 | 0.164772 |
