# Supplementary material for: Characterization and mapping of Dt1 locus which co-segregates with CcTFL1 for growth habit in pigeonpea
Source: Theor Appl Genet. 2017 May 24;130(9):1773–84. doi: 10.1007/s00122-017-2924-2 (PMC5565653; doi:10.1007/s00122-017-2924-2)
Supplement: Supplementary file 3 — Supplementary material 3 (PPTX 653 kb) [file 122_2017_2924_MOESM3_ESM.pptx]

## Slide 1
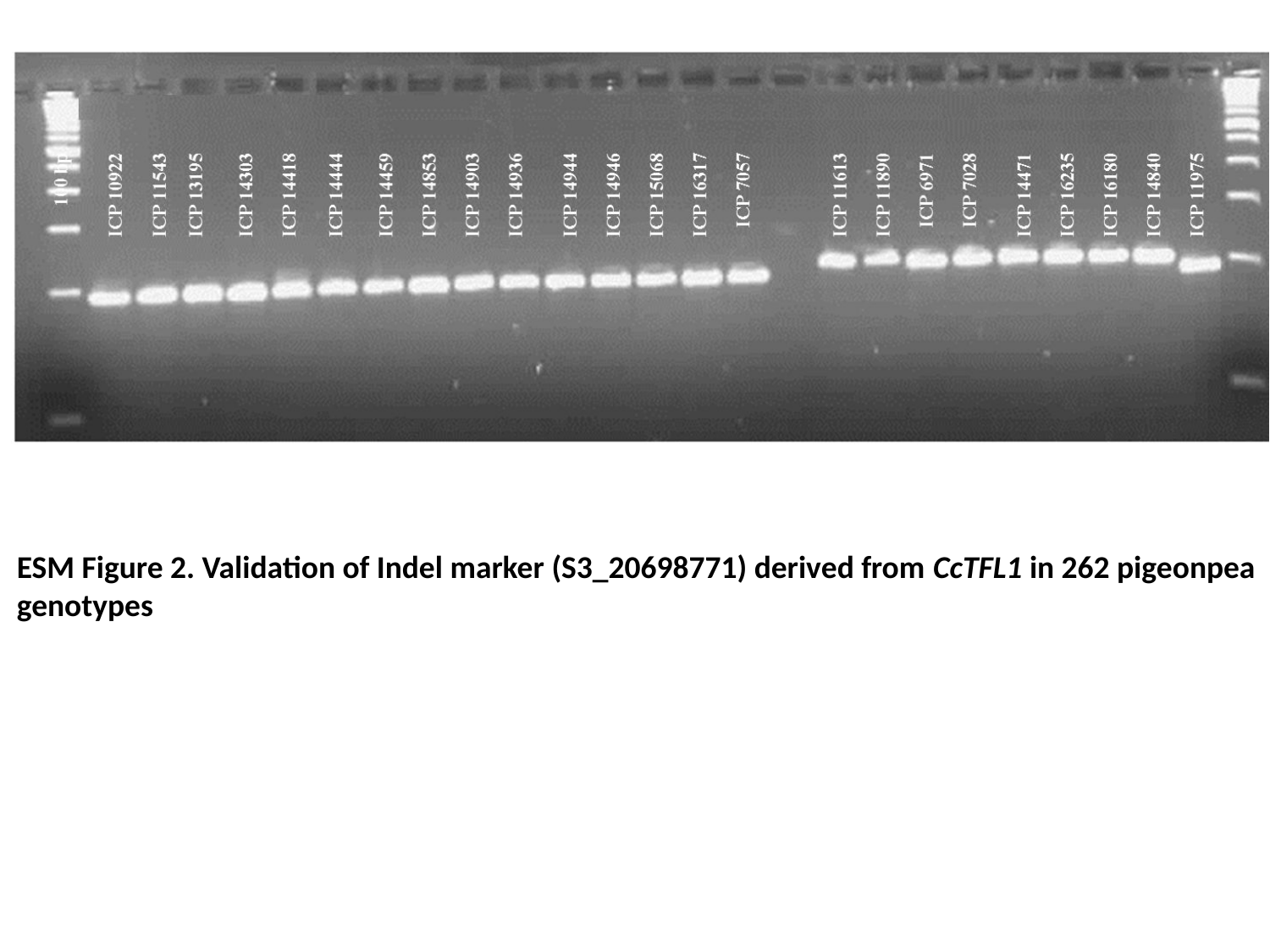

ESM Figure 2. Validation of Indel marker (S3_20698771) derived from CcTFL1 in 262 pigeonpea genotypes

## Slide 2
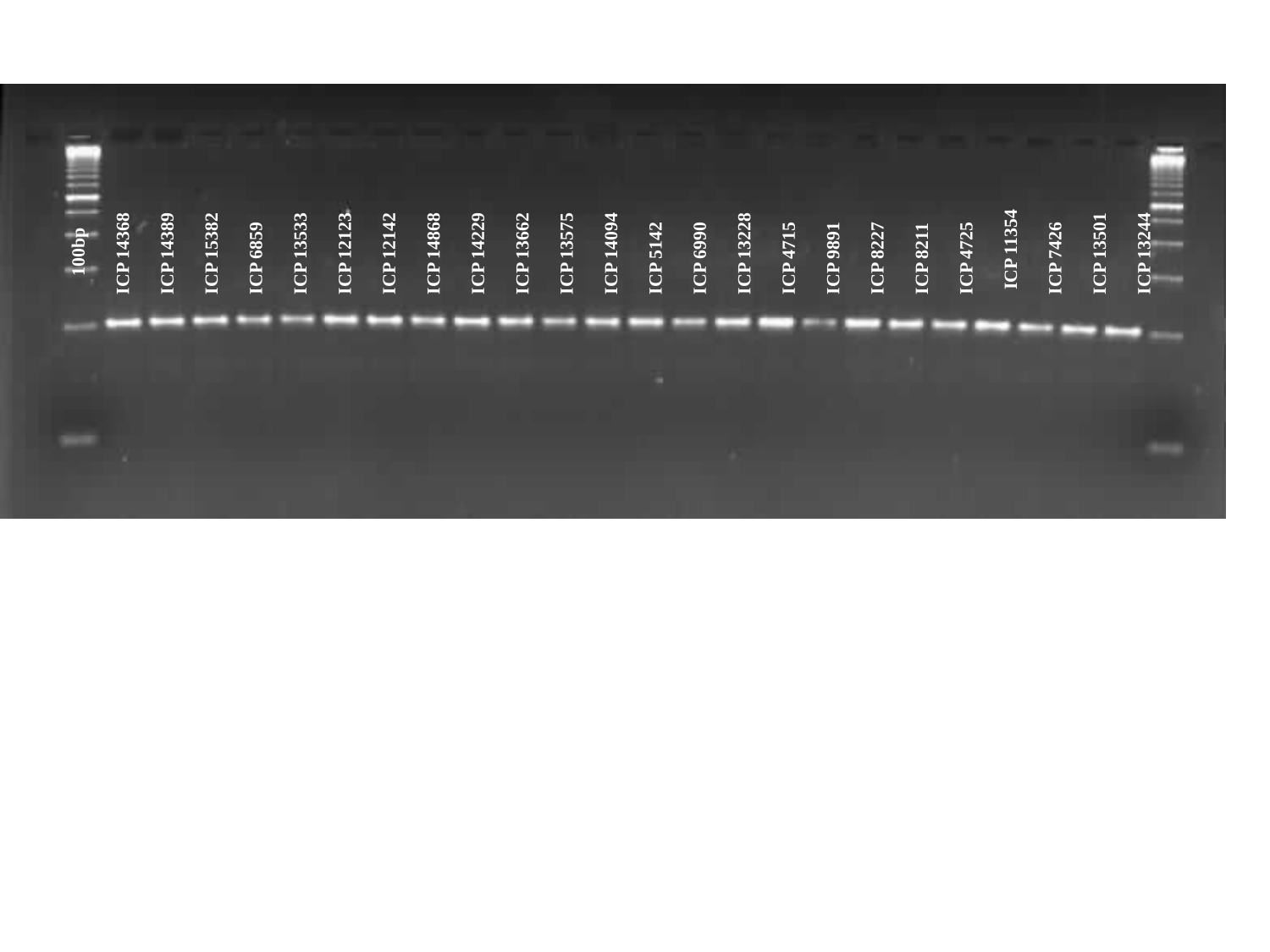

100bp ICP 14368 ICP 14389 ICP 15382 ICP 6859 ICP 13533 ICP 12123 ICP 12142 ICP 14868 ICP 14229 ICP 13662 ICP 13575 ICP 14094 ICP 5142 ICP 6990 ICP 13228 ICP 4715 ICP 9891 ICP 8227 ICP 8211 ICP 4725
 ICP 11354 ICP 7426 ICP 13501 ICP 13244

## Slide 3
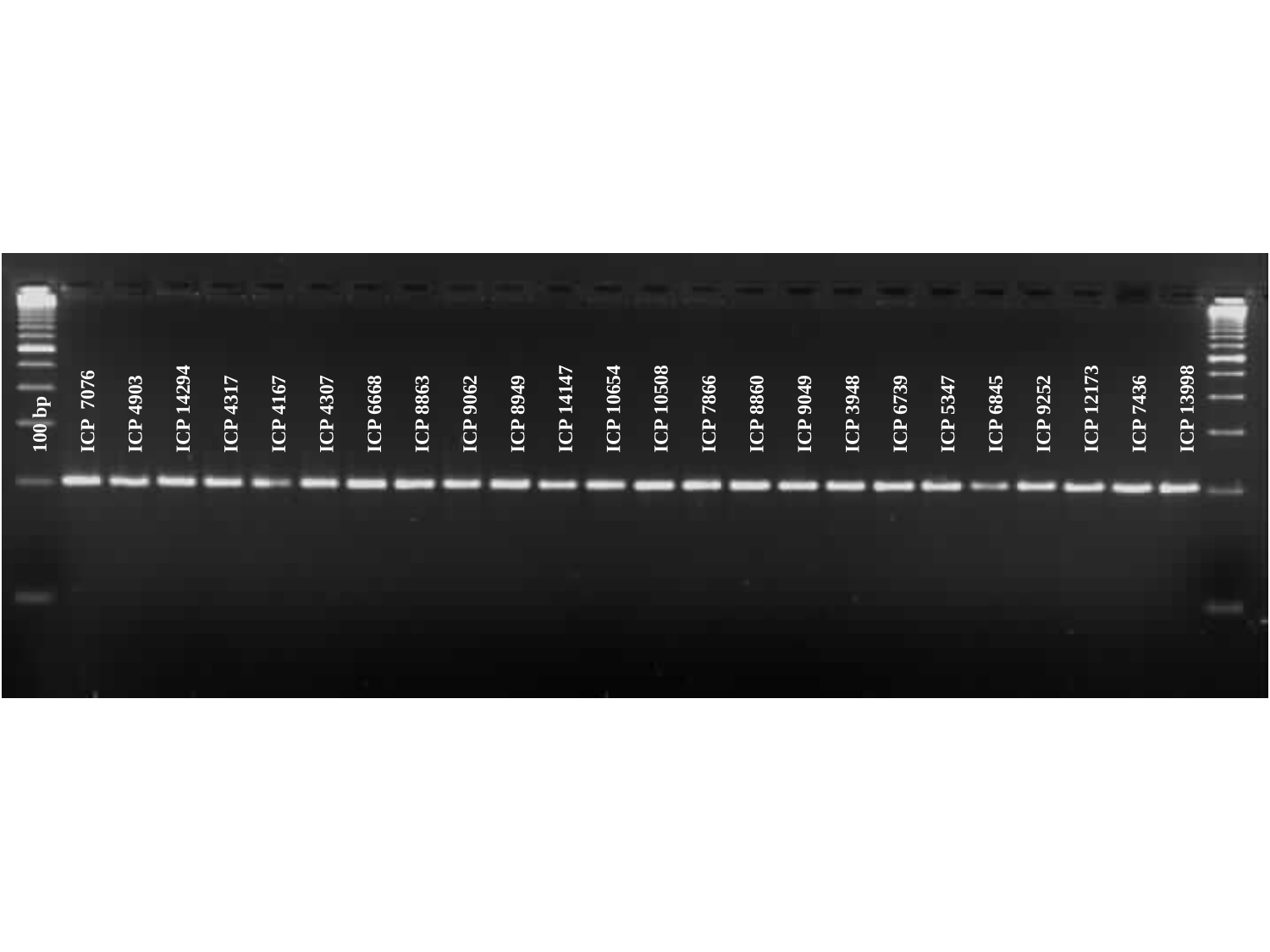

100 bp ICP 7076 ICP 4903 ICP 14294 ICP 4317 ICP 4167 ICP 4307 ICP 6668 ICP 8863 ICP 9062 ICP 8949 ICP 14147 ICP 10654 ICP 10508 ICP 7866 ICP 8860 ICP 9049 ICP 3948 ICP 6739 ICP 5347 ICP 6845 ICP 9252 ICP 12173 ICP 7436 ICP 13998

## Slide 4
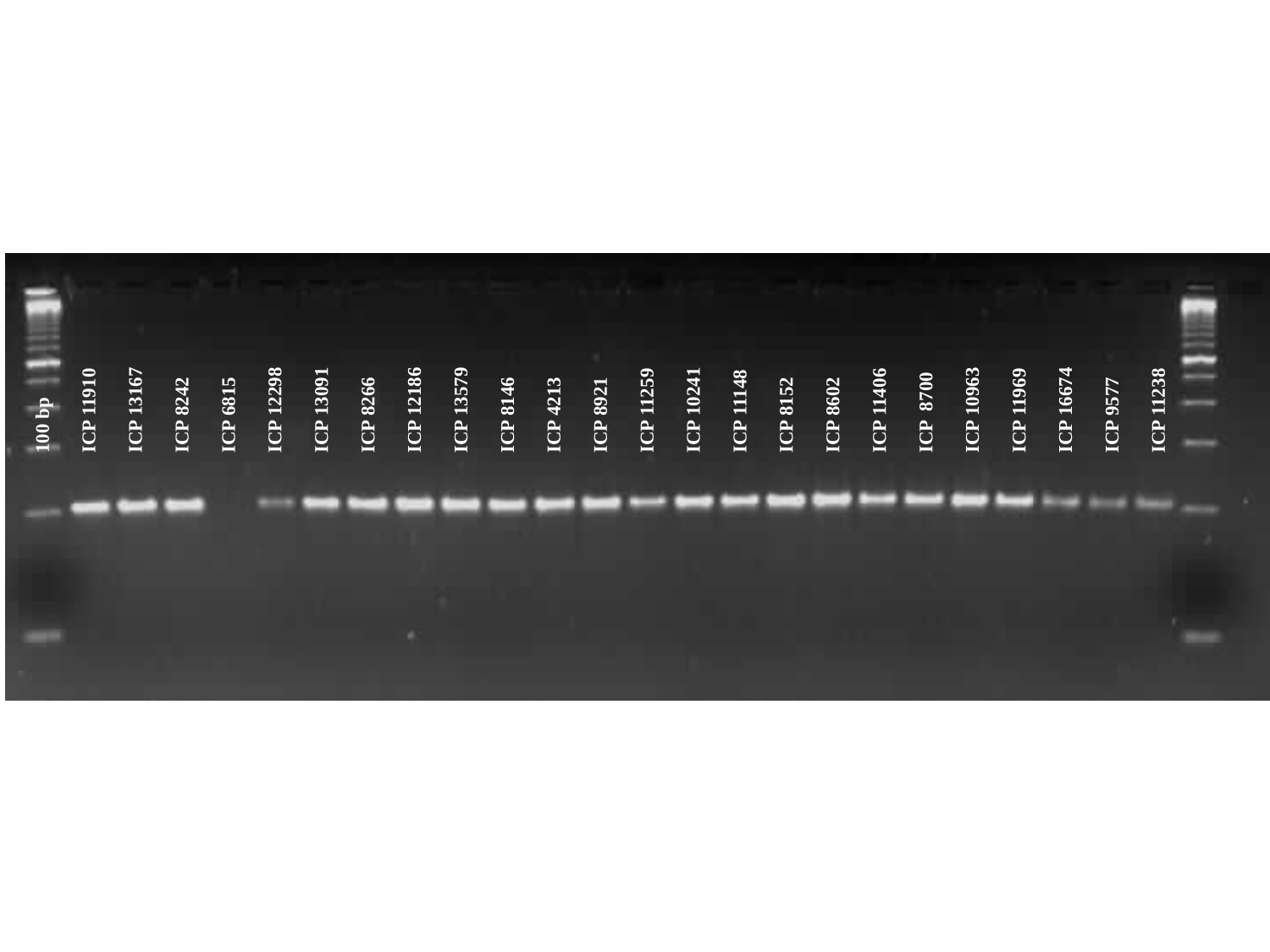

100 bp ICP 11910 ICP 13167 ICP 8242 ICP 6815 ICP 12298 ICP 13091 ICP 8266 ICP 12186 ICP 13579 ICP 8146 ICP 4213 ICP 8921 ICP 11259 ICP 10241 ICP 11148 ICP 8152 ICP 8602 ICP 11406 ICP 8700 ICP 10963 ICP 11969 ICP 16674 ICP 9577 ICP 11238

## Slide 5
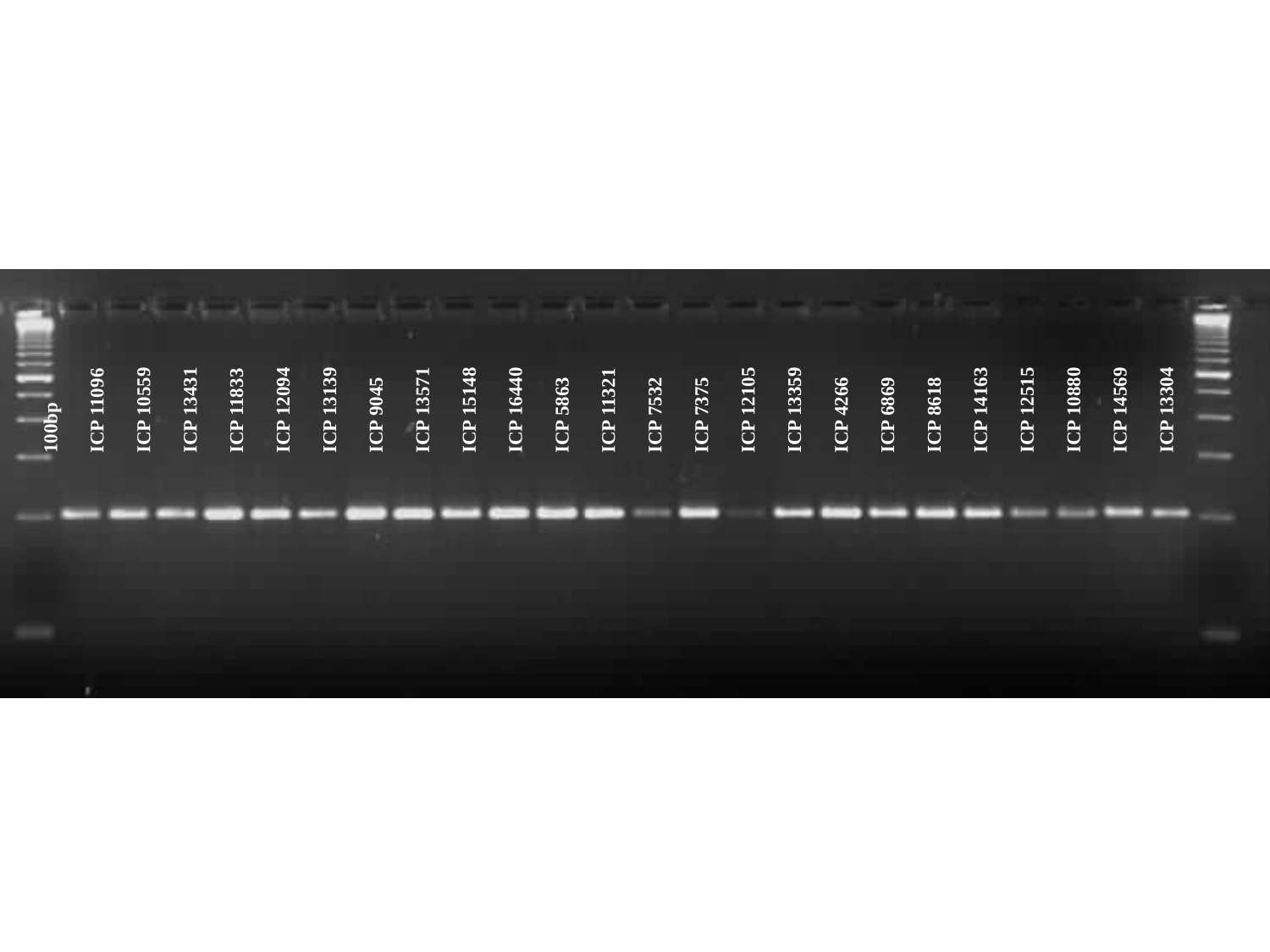

100bp
ICP 11096 ICP 10559 ICP 13431 ICP 11833 ICP 12094 ICP 13139 ICP 9045 ICP 13571 ICP 15148 ICP 16440 ICP 5863 ICP 11321 ICP 7532 ICP 7375 ICP 12105 ICP 13359 ICP 4266 ICP 6869 ICP 8618 ICP 14163 ICP 12515 ICP 10880 ICP 14569 ICP 13304

## Slide 6
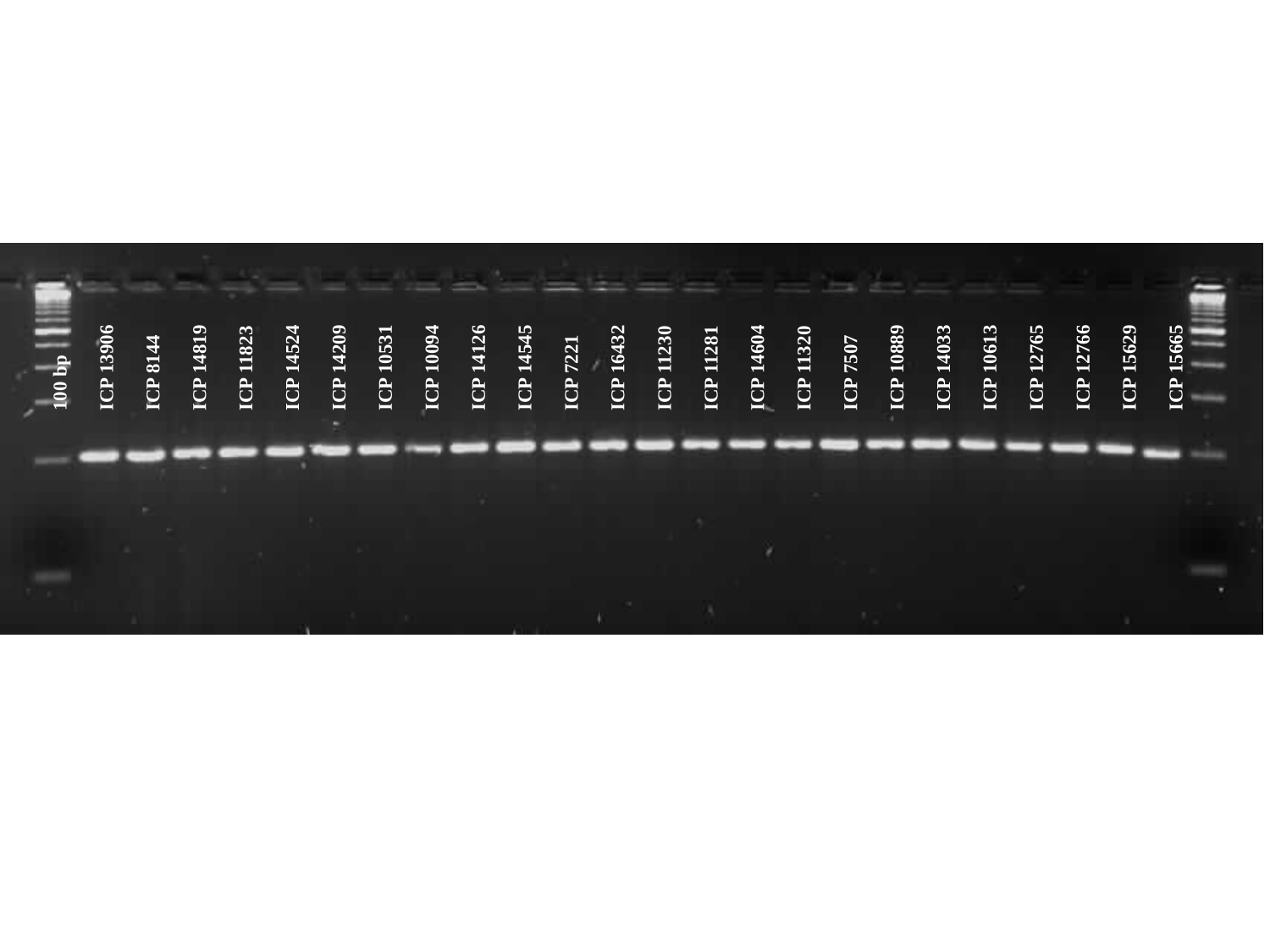

100 bp ICP 13906 ICP 8144 ICP 14819 ICP 11823 ICP 14524 ICP 14209 ICP 10531 ICP 10094 ICP 14126 ICP 14545 ICP 7221 ICP 16432 ICP 11230 ICP 11281 ICP 14604 ICP 11320 ICP 7507 ICP 10889 ICP 14033 ICP 10613 ICP 12765 ICP 12766 ICP 15629 ICP 15665

## Slide 7
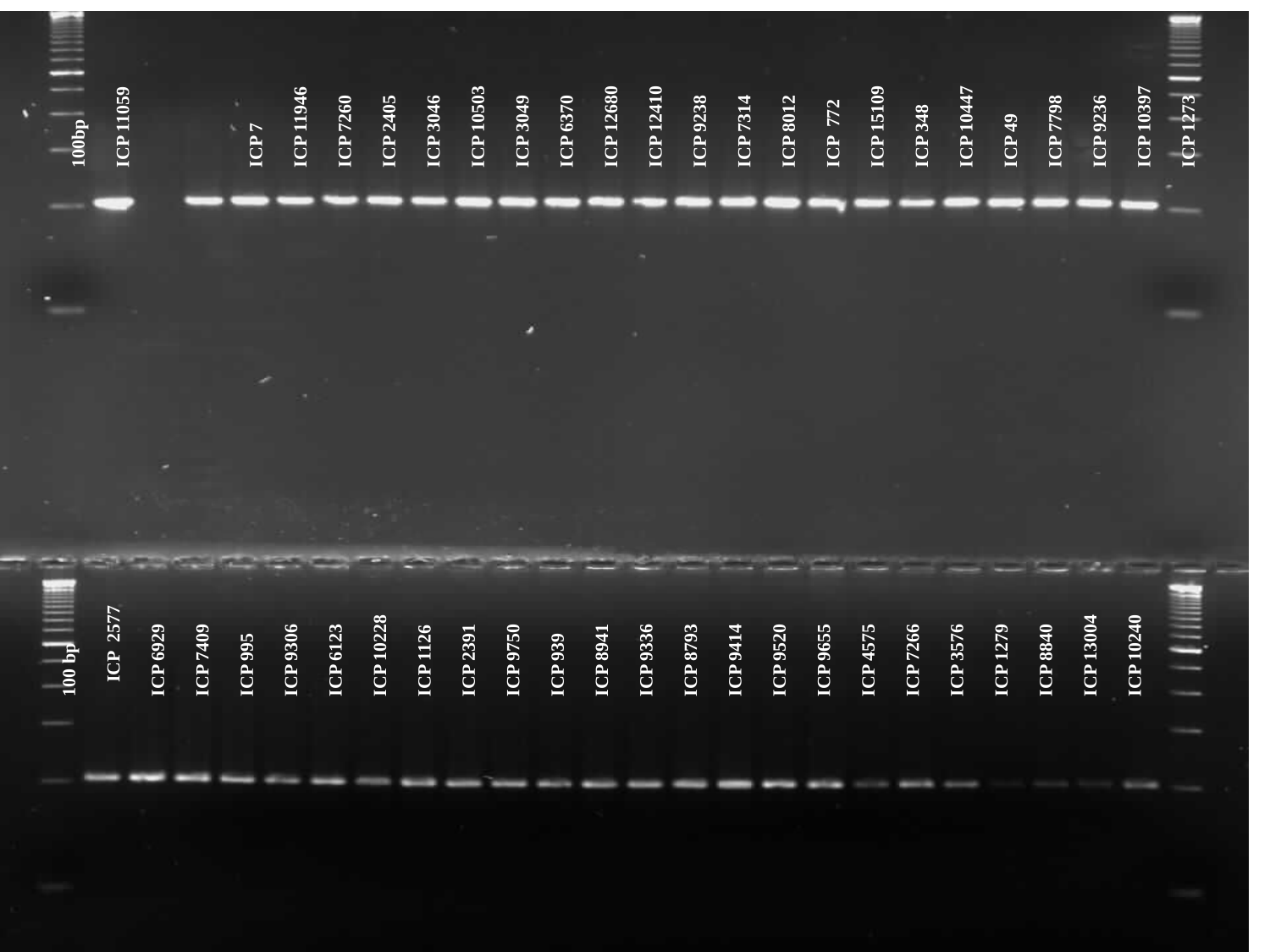

100bp ICP 11059
ICP 7
ICP 11946 ICP 7260 ICP 2405 ICP 3046 ICP 10503 ICP 3049 ICP 6370 ICP 12680 ICP 12410 ICP 9238 ICP 7314 ICP 8012 ICP 772 ICP 15109 ICP 348 ICP 10447 ICP 49 ICP 7798 ICP 9236 ICP 10397 ICP 1273
100 bp ICP 2577 ICP 6929 ICP 7409 ICP 995 ICP 9306 ICP 6123 ICP 10228 ICP 1126 ICP 2391 ICP 9750 ICP 939 ICP 8941 ICP 9336 ICP 8793 ICP 9414 ICP 9520 ICP 9655 ICP 4575 ICP 7266 ICP 3576 ICP 1279 ICP 8840 ICP 13004 ICP 10240

## Slide 8
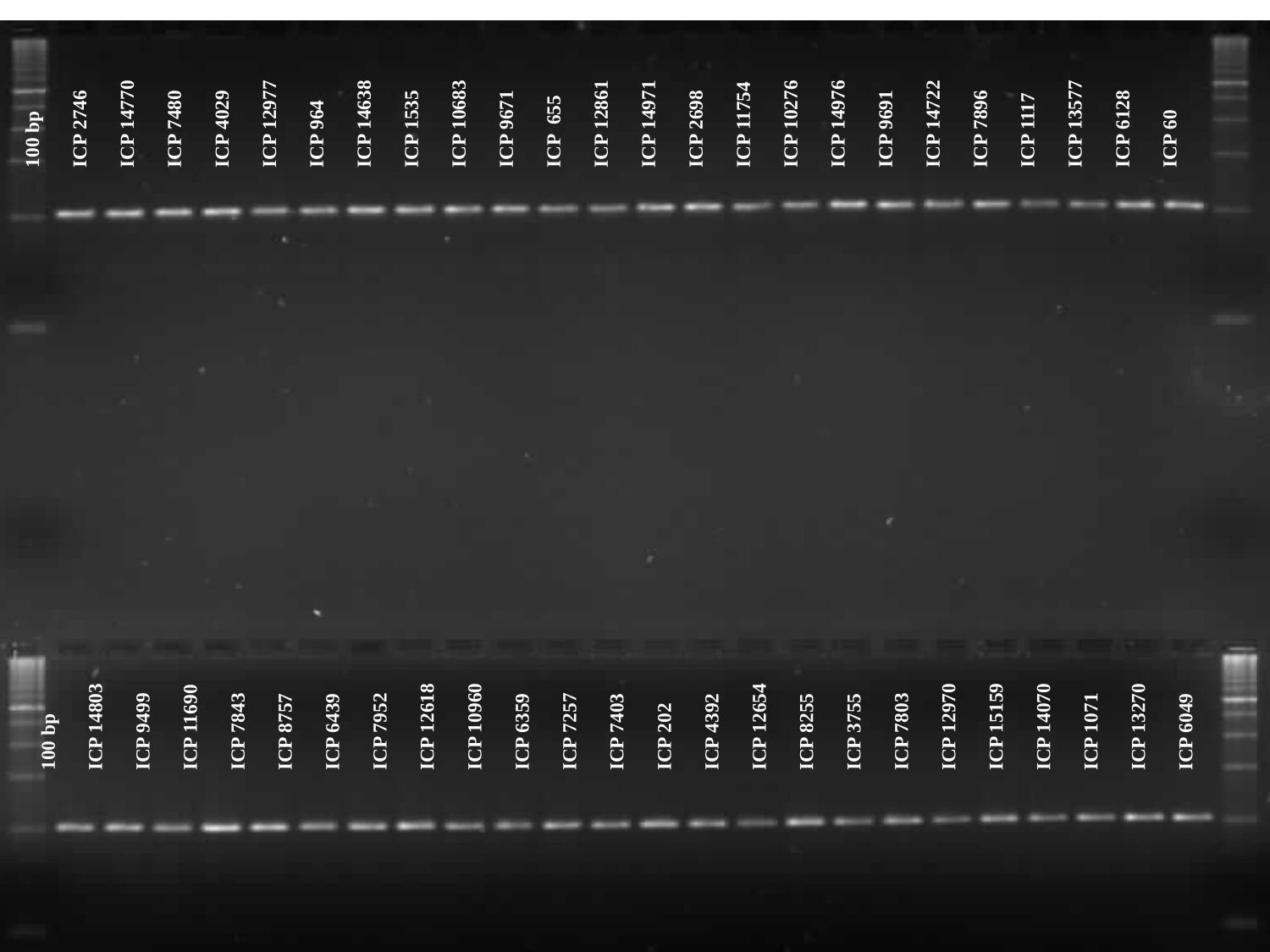

100 bp ICP 2746 ICP 14770 ICP 7480 ICP 4029 ICP 12977 ICP 964 ICP 14638 ICP 1535 ICP 10683 ICP 9671 ICP 655 ICP 12861 ICP 14971 ICP 2698 ICP 11754 ICP 10276 ICP 14976 ICP 9691 ICP 14722 ICP 7896 ICP 1117 ICP 13577 ICP 6128 ICP 60
100 bp ICP 14803 ICP 9499 ICP 11690 ICP 7843 ICP 8757 ICP 6439 ICP 7952 ICP 12618 ICP 10960 ICP 6359 ICP 7257 ICP 7403 ICP 202 ICP 4392 ICP 12654 ICP 8255 ICP 3755 ICP 7803 ICP 12970 ICP 15159 ICP 14070 ICP 1071 ICP 13270 ICP 6049

## Slide 9
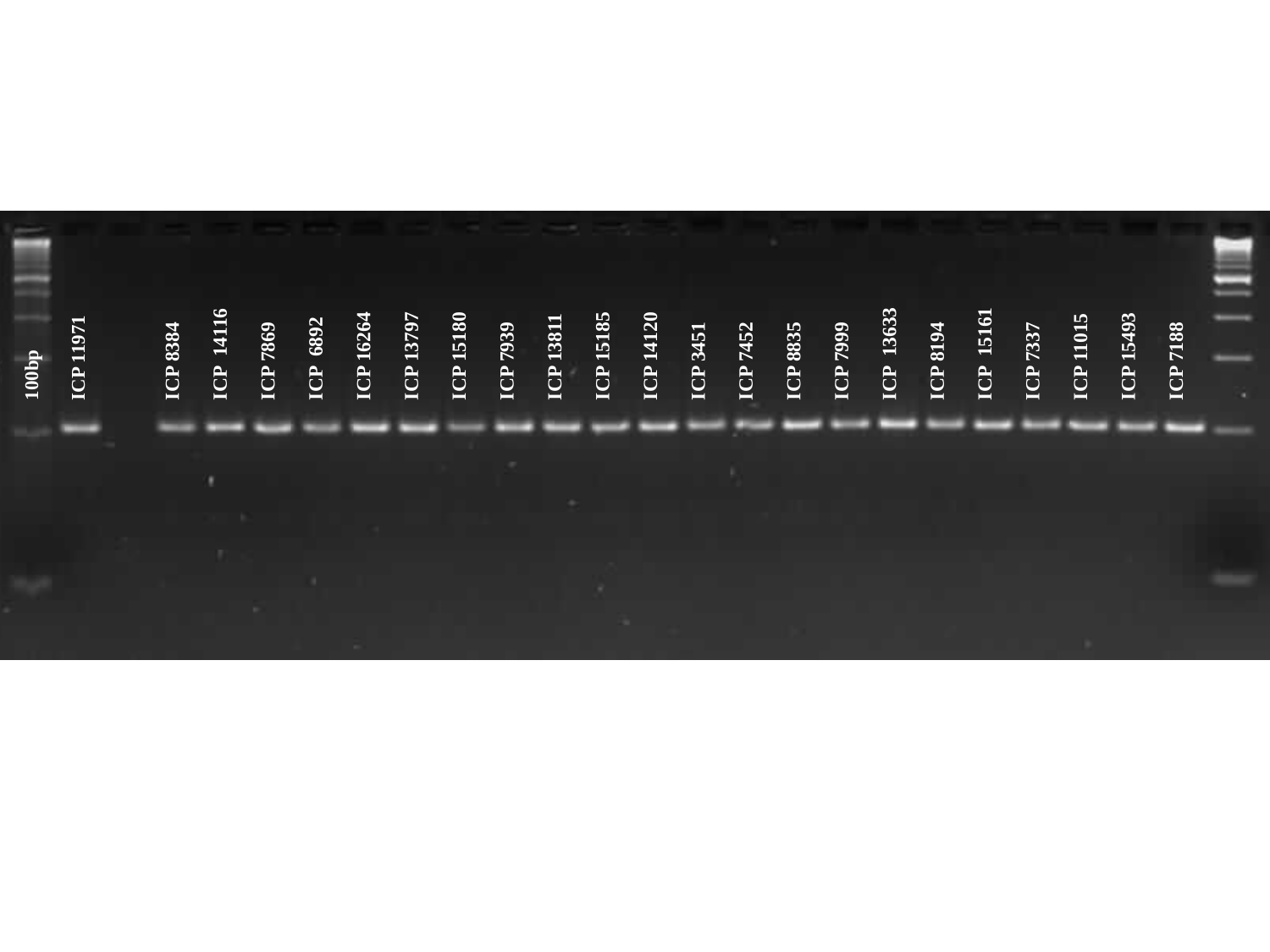

100bp ICP 11971
ICP 8384 ICP 14116 ICP 7869 ICP 6892 ICP 16264 ICP 13797 ICP 15180 ICP 7939 ICP 13811 ICP 15185 ICP 14120 ICP 3451 ICP 7452 ICP 8835 ICP 7999 ICP 13633 ICP 8194 ICP 15161 ICP 7337 ICP 11015 ICP 15493 ICP 7188
